# *S*-Nitrosylation in Organs of Mice Exposed to Low or High Doses of γ-Rays: The Modulating Effect of Iodine Contrast Agent at a Low Radiation Dose

**DOI:** 10.3390/proteomes3020056

**Published:** 2015-04-30

**Authors:** Fadia Nicolas, Changgong Wu, Salwa Bukhari, Sonia M. de Toledo, Hong Li, Masayuki Shibata, Edouard I. Azzam

**Affiliations:** 1 Department of Health Informatics, Rutgers School of Health Related Professions, Newark, NJ 07107, USA; E-Mail: nicolafa@shrp.rutgers.edu; 2 Department of Biochemistry and Molecular Biology, Rutgers New Jersey Medical School, Newark, NJ 07103, USA; E-Mail: wuchanggong1@yahoo.com; 3 Department of Radiology, Rutgers New Jersey Medical School, Newark, NJ 07103, USA; E-Mail: salwa.bukhari@gmail.com; 4 Department of Radiology, Rutgers New Jersey Medical School, Newark, NJ 07103, USA; E-Mail: detolesm@njms.rutgers.edu; 5 Department of Biochemistry and Molecular Biology, Rutgers New Jersey Medical School, Newark, NJ 07103, USA; E-Mail: liho2@njms.rutgers.edu; 6 Department of Health Informatics, Rutgers School of Health Related Professions, Newark, NJ 07107, USA; 7 Department of Radiology, RUTGERS New Jersey Medical School, Newark, NJ 07103, USA

**Keywords:** ionizing radiation, radiation sensitivity, *S*-nitrosylation, low- and high-dose γ ray responses, radiodiagnostic procedures, network analyses in brain

## Abstract

The covalent addition of nitric oxide (NO^•^) onto cysteine thiols, or *S*-nitrosylation, modulates the activity of key signaling proteins. The dysregulation of normal *S*-nitrosylation contributes to degenerative conditions and to cancer. To gain insight into the biochemical changes induced by low-dose ionizing radiation, we determined global *S*-nitrosylation by the “biotin switch” assay coupled with mass spectrometry analyses in organs of C57BL/6J mice exposed to acute 0.1 Gy of ^137^Cs γ-rays. The dose of radiation was delivered to the whole body in the presence or absence of iopamidol, an iodinated contrast agent used during radiological examinations. To investigate whether similar or distinct nitrosylation patterns are induced following high-dose irradiation, mice were exposed in parallel to acute 4 Gy of ^137^Cs γ rays. Analysis of modulated *S*-nitrosothiols (SNO-proteins) in freshly-harvested organs of animals sacrificed 13 days after irradiation revealed radiation dose- and contrast agent-dependent changes. The major results were as follows: (i) iopamidol alone had significant effects on *S*-nitrosylation in brain, lung and liver; (ii) relative to the control, exposure to 0.1 Gy without iopamidol resulted in statistically-significant SNO changes in proteins that differ in molecular weight in liver, lung, brain and blood plasma; (iii) iopamidol enhanced the decrease in *S*-nitrosylation induced by 0.1 Gy in brain; (iv) whereas a decrease in *S*-nitrosylation occurred at 0.1 Gy for proteins of ~50 kDa in brain and for proteins of ~37 kDa in liver, an increase was detected at 4 Gy in both organs; (v) mass spectrometry analyses of nitrosylated proteins in brain revealed differential modulation of SNO proteins (e.g., sodium/potassium-transporting ATPase subunit beta-1; beta tubulins; ADP-ribosylation factor 5) by low- and high-dose irradiation; and (vi) ingenuity pathway analysis identified major signaling networks to be modulated, in particular the neuronal nitric oxide synthase signaling pathway was differentially modulated by low- and high-dose γ-irradiation.

## 1. Introduction

Life on Earth has evolved in an environment of background radiation. Human and non-human biota are constantly exposed to ionizing radiation from natural sources, such as cosmic rays, radon decay products in the air and radionuclides in food and water. Moreover, since the discovery of X-rays in 1895, humans have been increasingly exposed to radiation from man-made sources, in particular medical diagnostic procedures, which have become key to modern medicine. Currently, medical imaging procedures constitute ~48% of the radiation exposure to the U.S. population, whereas in 1980, they accounted for 15% of the exposure [1]. Due to this dramatic rise, the effective dose per individual in the U.S. population has increased from 3.6 mSv in 1980 to 6.2 mSv in 2006 [1].

Exposures to high doses of ionizing radiation are known to cause immediate and delayed harmful biological outcomes, including toxic effects, persistent changes to DNA and perturbations in metabolic activity (reviewed in [2]). The debilitating health effects of such exposures are well characterized [3,4]. In contrast, the question of whether exposures to low doses of radiation also induce significant human health risks is a subject of debate and remains controversial [5,6]. In its BEIR VII report [7] (Biological Effects of Ionizing Radiation VII), the National Academies of Sciences in the USA concluded that statistical limitations make it difficult to evaluate cancer risks in humans at doses below 100 mSv. In effect, cohorts of 10^9^ and 5 × 10^6^ individuals would be required to detect excess risk of health hazards following exposures to 1 or 10 mSv, respectively [7]. Notably, doses in this range are received during mammography (~2.5 mSv), positron emission tomography (~3.7 mSv), technetium-99 cardiac scans (~4.4 mSv), other radiological procedures and may be received during occupational activities [1].

Due to the limitations in the statistical power of current human epidemiological studies in determining health risks from exposures to low-dose ionizing radiation, mechanistic laboratory studies have been judged to be essential for understanding the biological effects and to help evaluate health risks at low doses [7]. Animal or tissue culture studies permit tight control of many variables, which can contribute to clearer interpretations of human epidemiological studies and potentially to a reduction in the uncertainty of predicting adverse health risks of exposures to low-dose radiation [8,9].

To gain insight into the molecular and biochemical events underling the cellular responses at low doses of ionizing radiation, we and others have examined changes in gene expression at the levels of mRNA [10,11,12,13], microRNA [14,15] and protein [16,17,18] in rodent and human cells. Here, we build on these studies, but characterize post-translational modification of proteins, an area that is under-studied in the radiation sciences [19]. We used an *in vivo* approach and examined changes in *S*-nitrosylation in organs of young adult C57BL/6J mice at two weeks following exposure to low (0.1 Gy) or high (4 Gy) acute doses of ^137^Cs γ-rays delivered to the whole body. The goal was to explore whether similar or different biochemical changes occur following low- or high-dose irradiation. The outcome could have a bearing on an improved understanding of adaptive responses that have been reported to be induced following exposure to low doses of sparsely ionizing radiations, such as γ and X-rays [20]. Differences in the nature and/or extent of biochemical and molecular alterations resulting from the system responses to low- and high-dose radiation would suggest that the magnitude of modulation of biomolecular processes may not be necessarily proportional to radiation dose as implied by the linear-no-threshold model of radiation risk [21].

*S*-nitrosylation is the covalent addition of nitric oxide (NO^•^) onto the sulfur atom of a cysteine residue to form *S*-nitrosothiols (SNOs). At normal homeostatic concentration of NO^•^, nitrosylation is essential for normal protein function, including enzyme activation, protein-protein interactions and cellular trafficking [22,23]. On the other hand, aberrant *S*-nitrosylation has been implicated in oncogene activation [24] and in neurodegenerative diseases [25]. Among the many protein nitrosylation targets, several mediate the response to ionizing radiation, including caspases, HIF-1α, thioredoxin, manganese superoxide dismutase, NF-κB, p53 and Bcl-2 [23,26]. Despite the importance of *S*-nitrosylation in modulating protein function and location and the well-established knowledge that exposure to ionizing radiation triggers NO^•^ generation [27,28], the modification by radiation of specific cysteine residues *in vivo* is vastly unknown. In this study, we characterized *S*-nitrosylation following parallel exposures of mice to 0.1 Gy of ^137^Cs γ-rays, which mimics doses that are often received during radiographic scans (e.g., computed tomography), or 4 Gy, which may be received during radiotherapy or unintended exposures. To enhance our understanding of the effects of contrast agents used in imaging procedures, we analyzed SNO proteins in organs of mice exposed to 0.1 Gy in the presence or absence of iopamidol, which is commonly used in computed tomography (CT) procedures and other X-ray examinations. To gain insight into the change in signal transduction modulated by *S*-nitrosylation, we analyzed by mass spectrometry the *S*-nitroso proteome in brains of mice exposed to 0, 0.1 or 4 Gy of γ-rays. We used bioinformatics tools to analyze the results and to identify potentially modulated signaling pathways.

## 2. Experimental Section

Reagents: Chemical reagents were from Sigma (St. Louis, MO, USA), unless otherwise indicated. Acetonitrile (ACN) and HPLC-grade water were from J.T. Baker (Phillipsburg, NJ, USA). Formic acid was from EMD Chemicals (Merck KGaA, Darmstadt, Germany). Gel electrophoresis materials were from Bio-Rad (Hercules, CA, USA).

The contrast agent iopamidol (Isovue Multipack-370) was from Bracco Diagnostics Inc. (Princeton, NJ, USA). A 100-µL volume of Isovue 370 containing 37 mg of organically-bound iodine was injected retro-orbitally in the right eye of the mouse (~25 g) using a 27 G needle. Anesthesia was not used, and a one-minute interval separated the injection and the start of irradiation. The administered dose is slightly higher than the human dose, which would compensate for the higher metabolic activity of mice compared to humans [29] and, hence, would be more representative.

### 2.1. Animals and Radiation Treatment

The study protocol was reviewed and approved by the Institutional Animal Care and Use Committee of Rutgers University, New Jersey Medical School. The animal facility at the New Jersey Medical School is a specific pathogen-free barrier facility. Young adult (17-week-old) C57BL/6 mice were exposed to absorbed doses of 0.1 or 4 Gy of ^137^Cs γ-rays (661 keV) delivered at dose rates of 0.1 Gy/min and 1 Gy/min, respectively. The γ-ray dose that kills 50% of C57BL/6 mice in 30 days (LD_50/30_) is ~8 Gy to the whole body [30]. The radiation dose was administered to the whole body in a ventilated irradiator (J.L. Shepherd & Associates, Mark I, San Fernando, CA, USA) located within the vivarium. The mice were placed in a multi-chamber device (a single mouse per chamber) on a rotating platform to ensure uniform exposure. Mice in each group were equipped with individual dosimeters to verify the delivered dose (Mirion Technologies, Irvine, CA, USA). The average absorbed doses were not statistically different among the individual mice in each experimental group. Sham irradiated mice (0 Gy) were placed in the irradiator for 1 min, but the ^137^Cs source was not activated.

The experimental groups (5 mice/group) were as follows: (1) sham-irradiated without contrast agent; (2) sham-irradiated with contrast agent; (3) 0.1 Gy-irradiated without contrast agent; (4) 0.1 Gy-irradiated with contrast agent; and (5) 4 Gy-irradiated without contrast agent. The mice were placed in sterile cages immediately after irradiation to simulate reverse isolation. The food (sterile Purina rodent chow) and sterile drinking water were given *ad libitum*. The animals were sacrificed for analyses by CO_2_ asphyxiation at 13 days after irradiation, a time at which immediate DNA damage and oxidizing lesions to other macromolecules due to direct γ-irradiation would have been repaired, rendered permanent or degraded [31]. All of the animals had survived the treatment at the time of sacrifice. Immediately prior to asphyxiation, blood samples were collected by cardiac puncture and dispensed in anticoagulant-treated tubes. The plasma was separated from blood cells by centrifugation (1500× *g*, 10 min, 4 °C).

### 2.2. Biotin Switch Analysis of Protein Nitrosylation

A modified biotin switch technique [32] was used to identify nitrosylated proteins and their nitrosylation sites. In brief, whole mouse organs (brain, lung, liver) were minced, homogenized and lysed in lysis/blocking buffer (LB, 50 mM Tris, pH 7.5, 150 mM NaCl, 1% Triton^TM^ X-100, 2.5% SDS, 1 mM EDTA, 0.1 mM neocuproine and 50 mM methyl-methane thiosulfate (MMTS)). The lysis/blocking buffer was supplemented with a protease inhibitor cocktail, and the samples were frequently vortexed at 50 °C for 30 min. In the case of plasma, the lysis/blocking buffer (without MMTS) was added, and the protein concentration was measured by the BCA method and adjusted to 1 µg/µL. Free thiols were then blocked by adding 50 mM MMTS, and the samples were frequently vortexed at 50 °C for 30 min. Excess MMTS from the various samples was removed by cold acetone precipitation. The protein pellets were washed with 80% ice cold acetone and reconstituted in HENS buffer (100 mM Hepes (pH 7.8), 1 mM EDTA, 0.1 mM Neocuprine, 1% SDS w/v) containing 0.2 mM *N*-(6-(biotinamido)hexyl)-3'-(2'-pyridyldithio)-propionamide (biotin-HPDP) (Pierce, Rockford, IL, USA) with 10 mM ascorbate. The reaction mixture was incubated in the dark for 1 h at room temperature. Excess reagents were removed by cold acetone precipitation. The protein pellets were solubilized in non-reducing SDS-PAGE loading buffer (100 mM Tris, pH 6.8, 2% SDS, 15% glycerol, 0.01% bromophenol blue) for Western blotting or in resuspension buffer (RB; 50 mM Tris, pH 7.5, 150 mM NaCl, 1% Triton^TM^ X-100, 0.5% SDS) for immunoprecipitation, as described below. For Western blotting, 15-μg aliquots of protein were separated using non-reducing SDS-PAGE and transferred onto nitrocellulose membrane. The biotinylated proteins were reacted with an anti-biotin antibody (Vector Laboratories, Burlingame, CA, USA) and visualized with enhanced chemiluminescent substrate (PerkinElmer, Waltham, MA, USA).

### 2.3. Immuno-Precipitation and Detection of S-Nitrosylated Proteins

Proteins modified by the biotin switch assay were precipitated in acetone and dissolved in RB. Protein concentrations were determined by the BCA method. Biotinylated proteins (500 µg) in 500 µL RB were diluted with 500 µL PBS and mixed with 50 μL of streptavidin-agarose beads (Pierce, Rockford, IL, USA). The mixture was incubated for 1 h at 25 °C with agitation. The beads were washed 5× with 1 mL of PBS, suspended in 2× SDS-PAGE loading buffer and heated at 100 °C for 5 min. For detection of specific nitrosylated proteins, supernatant proteins were separated by SDS-PAGE and transferred onto a nitrocellulose membrane. The membranes were blocked with 5% milk and reacted with specific antibodies to confirm the mass spectrometry results. After incubation with a specific secondary antibody conjugated with horseradish peroxidase, protein bands were detected by an enhanced chemiluminescence system from GE Healthcare (Amersham, UK). Luminescence was determined by exposure to X-ray film, and densitometry analysis was performed with an EPSON scanner and National Institutes of Health ImageJ software (NIH Research Services Branch, Bethesda, MD, USA).

### 2.4. Analysis of Nitrosylated Proteins and Peptides by Mass Spectrometry

Mass measurement of *S*-nitrosylated proteins and peptides in brain tissues from mice exposed to 0, 0.1 or 4 Gy was performed on an LTQ Orbitrap Velos Pro mass spectrometer (Thermo Fisher Scientific, Waltham, MA, USA) according to an optimized method developed in our laboratory [32]. The mass spectrometer is coupled with a Dionex Chromatography System equipped with an Ultimate^TM^ 3000 autosampler through a Proxeon nano-electrospray ion source (Thermo Fisher Scientific, West Palm Beach, FL, USA). Mass spectrometry (MS) analyses were performed via direct infusion, unless stated otherwise. For identification of nitrosylated cysteines, biotinylated proteins were recovered with 8 M urea following acetone precipitation of the proteins from the biotin switch assay. The proteins were diluted 10-fold with 50 mM NH_4_HCO_3_ and digested with trypsin (1:30 w/w enzyme:protein ratio) at 37 °C overnight. The resulting peptides were loaded onto an avidin cartridge (ICAT kit from ABI) for enrichment of the biotinylated peptides. After washing the cartridge to remove unmodified peptides with 2 mL of PBS (pH 7.2) and 1 mL of a solution containing 50 mM ammonium bicarbonate and 20% methanol (pH 8.3), the biotinylated peptides were eluted with 30% ACN and 0.4% trifluoroacetic acid (TFA), dried in a SpeedVac concentrator and suspended in 2% ACN and 0.1% TFA. For LC/MS/MS analysis, the biotinylated peptides were first separated by Dionex UltiMate^®^ 3000 reversed phase liquid chromatography (RPLC, capillary PepMap 100 column, 75 μM × 150 mm, 3 µM, 100 Å, C_18_, Dionex, Sunnyvale, CA, USA). The eluted peptides were analyzed, and MS spectra (*m/z* 400–1900) were acquired in the positive ion mode. Argon was used as the collision gas. The collision energy was set from 16–60 V, depending on the precursor ion charge state and mass. The MS/MS spectra were acquired in the data-dependent analysis mode, in which the three most abundant precursors with two to five charges from each MS survey scan were selected for fragmentation. The peak lists were generated by ProteinLynx (v2.1, Waters, Milford, MA, USA) into files in PKL format. Database searches were performed with Mascot (2.4.1, Matrix Science, Boston, MA, USA) as a search engine against all of the mouse protein sequences in the UniRef100 protein database (downloaded on 24 January 2014). The following search parameters were used: trypsin was selected as the enzyme with 1 missed cleavage, mass tolerance of 100 ppm for MS and 0.6 Da for MS/MS, MMTS-modified and biotin-HPDP-modified cysteine and methionine oxidation were set as variable modifications. For MS/MS identification of the peptide’s nitrosylation site, we set a Mascot score threshold of at least 34, which corresponded to a *p*-value of 0.05 or better, the spectra were manually validated for post-translational modifications (PTMs). The false discovery rate was calculated to be <0.5% according to Peng *et al.* [33].

### 2.5. Ingenuity Pathway Analysis

To elucidate the cell signaling pathways of SNO-proteins in brain of mice treated with γ radiation, we used Ingenuity Pathway Analysis (IPA) [34]. The program uses a knowledge base derived from the literature to relate the proteins to each other, by direct and indirect interactions. Identity of the proteins and their expression values were uploaded onto IPA, and canonical pathways and molecular interaction networks were generated based on the knowledge stored in the Ingenuity Pathway Knowledge Base. A ratio of the number of metabolites that map to the canonical pathway divided by the total number of molecules that map to the pathway is displayed in the Results Section. Fisher’s exact test was used to calculate a *p*-value determining the probability that the association between the metabolites and the canonical pathway was explained by chance alone.

### 2.6. Protein Classification and Functional Analysis

The biological functions of identified SNO-proteins were grouped according to the annotation with the PANTHER (Protein Analysis Through Evolutionary Relationships) classification system, which relates protein sequences to the evolution of specific protein functions and biological roles [35].

### 2.7. Statistical Analysis

Fold changes in the level of nitrosylated proteins detected in Western blots were expressed as the mean ± standard error (SEM). Statistical analysis was performed using two-tailed unpaired Student’s *t*-test with Excel. Differences were considered significant for *p* < 0.05.

## 3. Results

### 3.1. The Effect of Low and High Doses of *γ*-Rays on Global Changes in S-Nitrosylation in Organs of Irradiated Mice: The Impact of Iodine Contrast Agent on the Low-Dose Radiation Response

**Figure 1:**
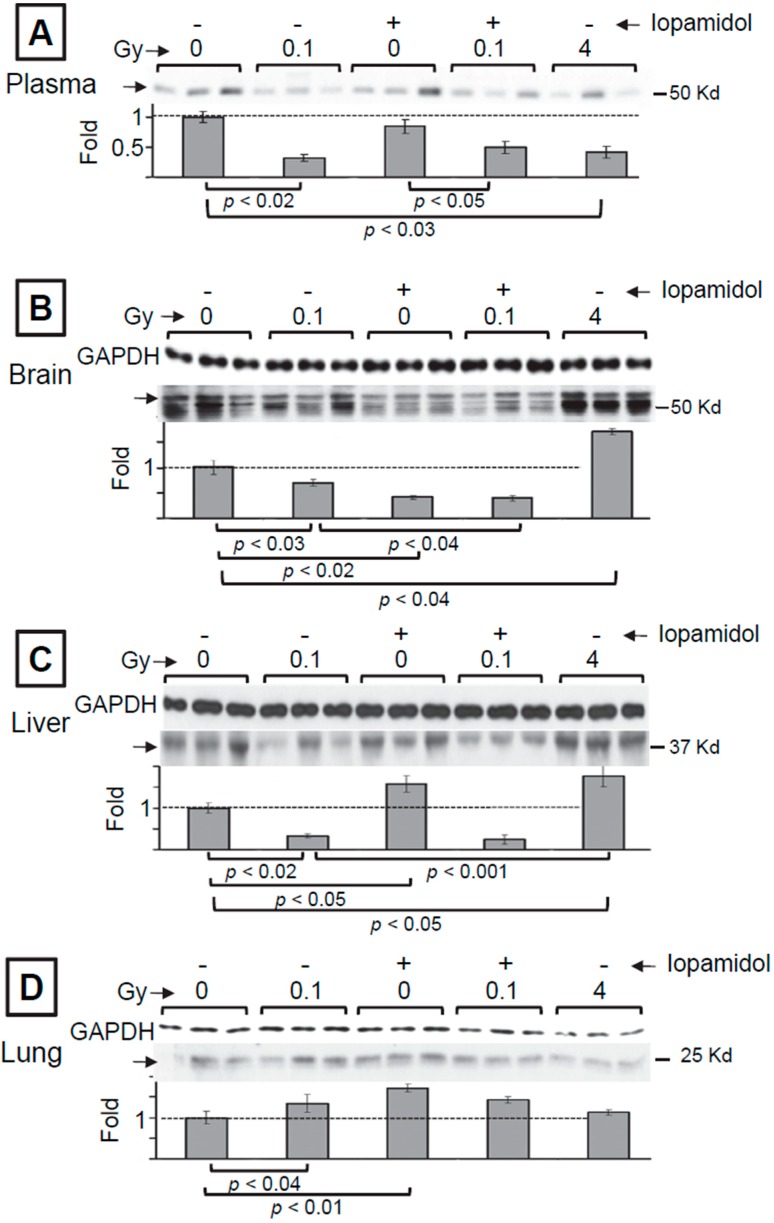
Modulation of *S*-nitrosylation, *in vivo*, by ionizing radiation and/or radiocontrast agent. Western blot analyses, following the biotin switch assay, of nitrosylated proteins from plasma (**A**), brain (**B**), liver (**C**), and lung (**D**) of C57 of C57Bl/6J mice exposed 13 days earlier to ^137^Cs γ-rays in the presence or absence of iopamidol. Proteins from mouse organs were freshly extracted and subjected to the biotin switch assay. The biotinylated proteins were detected with anti-biotin antibody. Protein aliquots (15 µg), before enrichment, were used as the input standard, and the expression level of GAPDH was used as the loading control. In the case of plasma from circulating blood, staining of the membrane with Ponceau S Red (not shown) indicated equal loading.

Deregulation of *S*-nitrosylation has been associated with degenerative diseases [36]. Further, recent findings have suggested that exposures to moderate and even low doses of ionizing radiation can induce degenerative conditions [37], including neurodegeneration and neuroinflammation [38]. To characterize the dependence of nitrosative stress on radiation dose, we investigated global *S*-nitrosylation by the ‘biotin switch’ assay in whole organs of irradiated mice. To this end, changes in the levels of SNO proteins with varying molecular weights in liver, lung, brain, as well as plasma were examined 13 days after exposure of 17-week-old C57BL/6J male mice to acute doses of 0, 0.1 or 4 Gy of ^137^Cs γ-rays. To gain understanding of the modulating effect of contrast agents used in radiodiagnostic procedures, mouse exposure to 0 or 0.1 Gy was carried out in the presence or absence of iopamidol, an iodinated agent that was delivered intravenously immediately prior to irradiation. The biotin switch assay results in Figure 1 describe representative radiation dose-dependent changes in the levels of nitrosylated proteins extracted from freshly-harvested organs of three individual mice. The Western blot analyses in the various panels show that relative to the control, exposure to 0.1 Gy resulted in prominent changes in the levels of SNO proteins in plasma, brain and liver. Interestingly, the decreases observed at 0.1 Gy in brain and liver are contrasted with increases in SNO proteins with a similar molecular weight (40–60 kDa) in brain and liver of mice exposed to 4 Gy. In plasma, decreases in similar magnitude to those observed at 0.1 Gy were detected at 4 Gy for proteins of the molecular weight range shown in (A) of Figure 1. In lung, comparable increases were detected at 0.1 and 4 Gy. The full range of changes in SNO proteins of different molecular weights is shown in Supplementary Figure S1.

Relative to control mice, administration of contrast agent alone or in combination with 0.1 Gy also resulted in modulation of *S*-nitrosylation in a few of the organs examined (Figure 1). The changes are highlighted in the autoradiograms for proteins of a specific size. In blood plasma, for proteins of MW in the range of 45–50 kDa, treatment with iopamidol alone or in combination with 0.1 Gy exposure did not induce an apparent effect. In contrast, in brain, iopamidol alone resulted in a greater decrease than induced by 0.1 Gy, but did not result in an additional effect when combined with 0.1 Gy. In lung, iopamidol triggered a significant increase in *S*-nitrosylation of proteins in the 50–60 kDa MW range. This increase was not affected when iopamidol treatment was combined with 0.1 Gy exposure. In liver, iopamidol treatment enhanced *S*-nitrosylation of proteins in the 50-kDa range. However, when iopamidol treatment was combined with exposure to 0.1 Gy, a decrease similar to that observed following irradiation alone was observed (see Supplementary Figure S1 for the full range of changes in SNO proteins with different molecular weights).

### 3.2. Mass Spectrometry Analysis of S-Nitrosylated Proteins in Brains of Irradiated Mice

**Figure 2:**
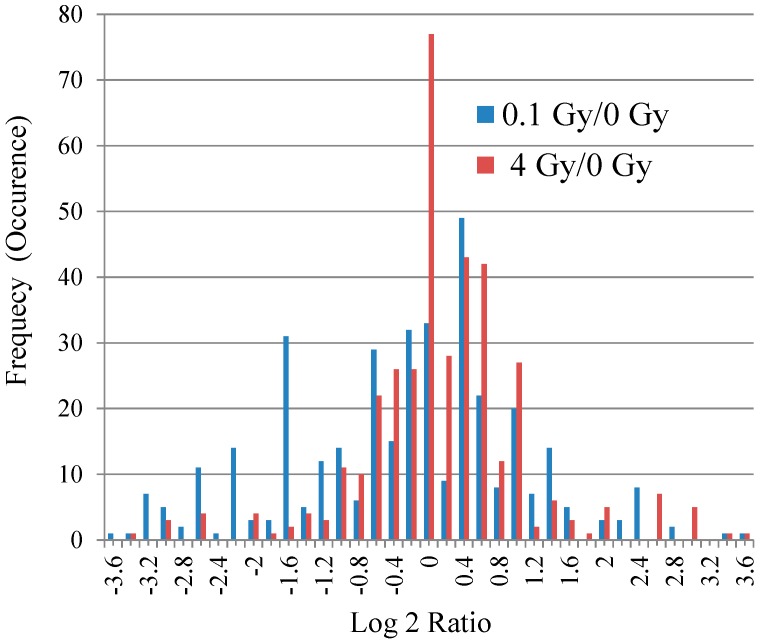
Modulation of the *S*-nitrosoproteome in brain of mice exposed to low- or high-dose ^137^Cs γ-rays. Changes in the levels of nitrosylated proteins detected by mass spectrometry analyses in brains of C57Bl/6J mice exposed 13 days earlier to acute doses of 0.1 Gy or 4 Gy of ^137^Cs γ rays. The X-axis is the relative level of nitrosylated proteins after a given radiation dose compared to the control (numbers shown represent the fold-change of nitrosylated proteins detected and expressed in the log2 ratio, where x indicates the interval between x − 0.1 and x + 0.1). The Y-axis indicates the frequency or number of occurrences in terms of the number of proteins. Blue and red bars indicate observations at 0.1 Gy and 4 Gy, respectively, compared to the control.

**Figure 3:**
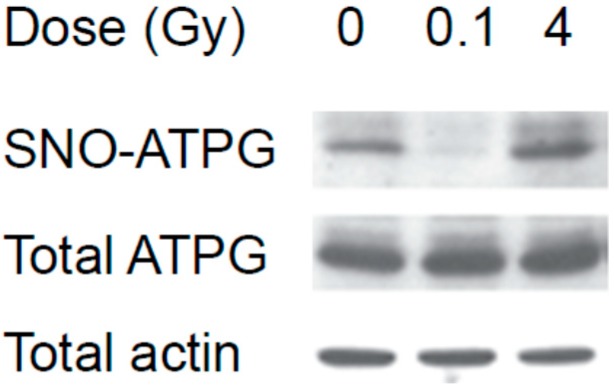
Western blot analyses of selected protein in brain of irradiated C57Bl/6J mice. Proteins from mouse brain were freshly extracted and submitted to the biotin switch assay. They were subsequently fractionated, immunoblotted, as described in the Experimental Section, and examined for the expression level of SNO- and total ATPG. Protein aliquots (15 μg) from each sample, before enrichment, were used as the input standard.

Exposures to ionizing radiation even at doses similar to those received during CT scans have been suggested to increase the risk of developing brain tumors [39]. Interestingly, the data in Figure 1 revealed differential changes in levels of SNO proteins (e.g., in the 40–60-kDa range) in brains of mice exposed to 0.1 or 4 Gy. To gain insight into the modulated proteins, we performed mass spectrometry (MS) measurements of *S*-nitrosylated proteins and peptides in lysates of whole brain of mice exposed 13 days earlier to 0, 0.1 or 4 Gy. To this end, equal mass aliquots of protein lysates from five different mice in each group were combined and submitted for the biotin switch assay and MS analyses. Spectral counts (Supplementary Table S1) revealed that 377 SNO proteins were detectable in at least one condition among the three conditions examined (0 Gy, 0.1 Gy and 4 Gy). Relative to the control, the majority of proteins from irradiated samples exhibited very small changes in nitrosylation, with most belonging to less than a two-fold increase or decrease (between −1 and +1 in the log2 ratio) (Figure 2). It is noteworthy that in the 4 Gy group, a large number of proteins (77 proteins) showed almost no change in the nitrosylation level from the control, with ~86% of the proteins showing less than a ±2-fold change (*i.e.*, within ±1 in the log2 ratio). Interestingly, a greater number of proteins showed decreased nitrosylation after low-dose radiation exposure, which is similar to what has been found in the immunoprecipitation experiments (Figure 1). Interestingly, the Western blot analyses in Figure 3, showing expression levels of both nitrosylated and total ATPG (ATP synthase subunit γ, mitochondrial), indicate that at least for some proteins, the change in level of nitrosylation is not due to the modulation of total protein expression level, but rather to regulation of *S*-nitrosylation.

**Figure 4:**
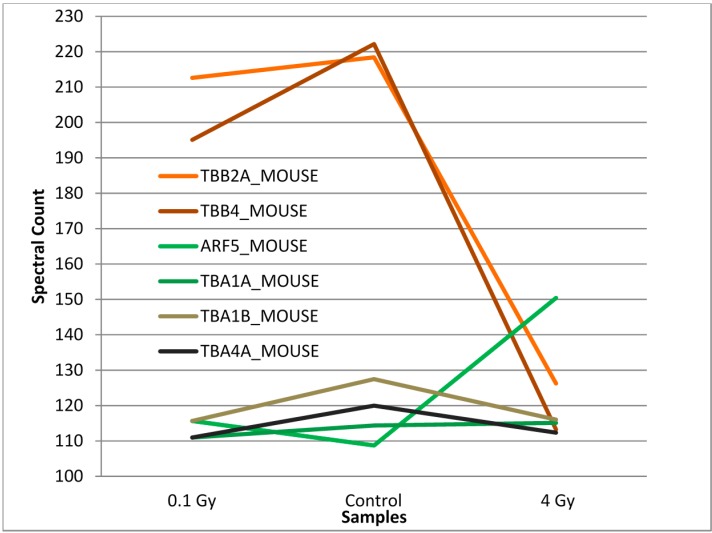
Differential effect of γ-ray dose on *S*-nitrosylation level. A comparison of spectral counts of SNO proteins (proteins with spectral counts above 80) showed differential modulation relative to the control of β tubulins in brain of mice exposed to 4 Gy, while no such effect was observed at 0.1 Gy. *S*-nitrosylation of ADP-ribosylation factor 5 (ARF5 shown in green) increased at 4 Gy.

The majority of the detected proteins had spectral counts of less than 10, and there were only small deviations among the three different conditions. Only a few proteins exhibited a spectral count above 80 regardless of the experimental conditions (Figure 4). Among these six proteins, tubulin betas (TBB4 and TBB2A) showed a slight decrease in spectral counts at 0.1 Gy compared to 0 Gy, while the magnitude of decrease was more enhanced at 4 Gy (Figure 4, line graph or, alternatively, the bar graph in Supplementary Figure S2). Compared to tubulin beta, no appreciable changes were observed for tubulin alphas (TBA1A, TBA1B, TBA4A) at both low and high doses compared to 0 Gy. One protein that showed almost no difference in spectral counts at 0.1 Gy, but a slight increase at 4 Gy, is ADP-ribosylation factor 5 (ARF5) (Figure 4). Another protein that exhibited a >80 spectral count is AT1B1 (sodium/potassium-transporting ATPase subunit β-1). Compared to the other six proteins, AT1B1 showed spectral counts of 25 and 20 at 0 Gy and 4 Gy, respectively, while the count rose to 120 at 0.1 Gy.

**Figure 5:**
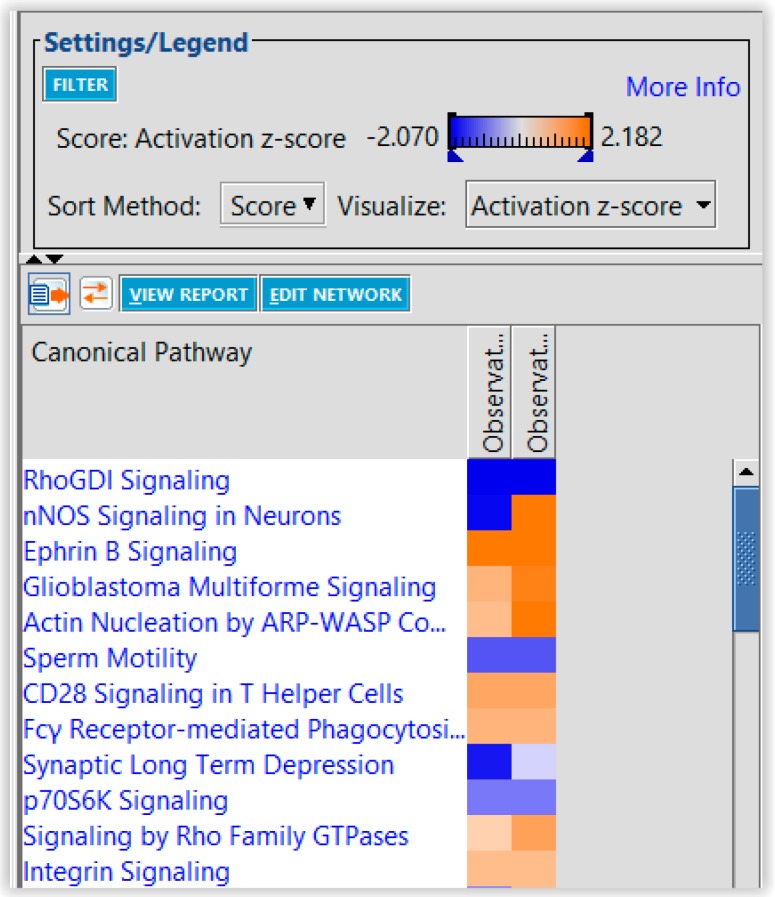
Heat map highlighting enriched canonical pathways in brains of mice that were exposed to ^137^Cs γ-rays. Data describing the fold change relative to the control in SNO proteins in brains of mice exposed, 13 days earlier, to 0.1 or 4 Gy were uploaded into Ingenuity Pathway Analysis (IPA). The different pathways enriched under each observation are shown. Whereas nNOS is apparently inhibited by 0.1 Gy (blue color), it is activated by 4 Gy (orange color).

The distribution of the 377 modulated SNO proteins detectable in at least one condition among the three conditions investigated (0 Gy, 0.1 Gy, 4 Gy) was examined by IPA [34]. The fold changes (0.1 Gy/0 Gy and 4 Gy/0 Gy) were uploaded into the IPA, and the generated top enriched pathways are illustrated in Figure 5. The activation score (or Z-score) for modulated SNO proteins ranged from −2.1 to 2.2, which is rather modest. Of particular interest, the data suggest that the neuronal nitric oxide synthase signaling pathway [40,41] is differentially modulated by low- and high-dose irradiation.

Gene ontology analyses by PANTHER [35] showed that the 377 proteins whose *S*-nitrosylation status has been modulated in our dataset are likely implicated in functions related to metabolic (229 proteins) and cellular processes (146 proteins). Within cellular processes, more than half (81 proteins) belong to cell communication and the cell cycle (40 proteins). It is interesting to note that the top network identified by the IPA belongs to cell to-cell signaling and interaction, nervous system development and function and neurological disease, due to the large number of molecules represented in our dataset.

## 4. Discussion

The roles and effects of *S*-nitrosylation in signal transduction are not as well characterized as other post-translational modifications [42,43]. However, recently, *S*-nitrosylation has gained increased attention, especially in neurobiology and brain research [25]. In particular, *S*-nitrosylation was shown to have an effect on phosphorylation, acetylation, ubiquitination, SUMOylation and redox modifications of proteins [44]. To enhance our understanding of the biochemical changes induced by low and high doses of γ-rays, we examined the levels of SNO proteins in organs of irradiated mice 13 days after irradiation. We also investigated the impact of iopamidol, a contrast agent used in radiological imaging procedures. Remarkably, relative to sham-treated controls, altered levels of SNO proteins were detectable long (13 days) after the radiation exposure or iopamidol treatments (Figure 1). However, when treatments with both agents were combined, the effect on the global level of SNO proteins detected by immunoblotting was not additive and seemed to reflect the impact of the irradiation. Iopamidol is a nonionic water-soluble chemical that rapidly clears from the body following administration [45]. Although it is generally well tolerated, studies have indicated that it can induce significant biological side-effects that become evident long after the exposure. Interestingly, spermatogenesis studies in mice have shown that contrast agents, including iopamidol, affect sperm head survival assayed 15–25 days following treatment [46]. Whether such persistent effects involve nitrosylation remains to be investigated. Interestingly, *S*-nitrosylation was shown to inhibit the activity of the mitogen-activated protein kinase that is involved in spermatogenesis [47].

Our mass spectrometry analyses of SNO proteins and peptides in brain of mice revealed that a group of 377 proteins showed changes in levels after whole body irradiation of the animals with 0.1 or 4 Gy. However, most of the proteins exhibited a small magnitude of change relative to sham-treated mice, with the majority belonging to less than a two-fold increase/decrease (Figure 2). Relative to the control, greater decreases in nitrosylation occurred at 0.1 Gy compared to 4 Gy exposure. Notably, the greater number of SNO proteins observed to be decreased at 0.1 Gy (Figure 2) is consistent with the decreases observed in the Western blot analyses in Figure 1 for proteins in the 40–60-kDa range. However, our MS analyses measured only the amounts of SNO proteins, and it is not possible to clearly decipher whether this is due to a change in *S*-nitrosylation activity or is a reflection of the change in native protein levels. Examining both the changes in general and *S*-nitroso proteomes at early (hours), intermediate (weeks) and late (months) time points after irradiation by sensitive and quantitative methods would enhance the understanding of the underlying mechanism. Nevertheless, the immunoblot analyses of ATPG (Figure 3) support the validity of the MS results for this protein (Supplementary Table S1) and suggest that the general changes in nitrosylation observed in our MS results are not due to the altered level of expression of the native proteins.

Following exposure to either 0.1 Gy or 4 Gy, AKT3 (RAC-γ serine/threonine-protein kinase) was one of the proteins with the highest fold increase, since no nitrosylated AKT3 was observed in control samples (Supplementary Table S1). AKT3 is a member of the PI3K/AKT signaling proteins that also include PTEN, whose nitrosylation was also found to be modulated by γ-rays in our dataset (Supplementary Table S1). Interestingly, *S*-nitrosylation of PTEN has recently been reported to act as an on-off system for PI3-kinase-Akt signaling. *S*-nitrosylation of PTEN by low concentrations of NO at Cys-83 inhibited its enzymatic activity [48] and consequently stimulated the downstream Akt cascade, which is essential for cell survival [49]. Therefore, our results add to the wealth of knowledge about phosphorylation events and the activation of signaling by Akt.

A salient finding of our study is that relative to the control, nitrosylated tubulin betas (TBB4 and TBB2A) were decreased as a function of radiation dose in brain, whereas nitrosylated tubulin alphas were essentially unchanged. Interestingly, the most recently-discovered post-translational modification of tubulin was the *S*-nitrosylation of α- and β-tubulins in the murine brain [50]. Three cysteine residues in α-tubulin and four in β-tubulin have been identified to be susceptible to this modification [51], and it has been reported that *S*-nitrosylation of tubulin alters its polymerization [52]. Importantly, dysfunction of the cytoskeleton has been associated with numerous neurodegenerative conditions, including Alzheimer’s and Parkinson’s disease [53].

Our results have also shown that nitrosylated ARF5 level was increased in brain of mice exposed to 4 Gy. The ARFs are a family of small guanine nucleotide-binding proteins known to play a role in vesicular trafficking and organelle structure by recruiting coat proteins, regulating phospholipid metabolism and modulating the structure of actin at membrane surfaces [54]. They influence actin assembly at the Golgi to facilitate vesicle fission. The exact functions of the ARF5 are unclear; however, some studies have indicated that it might have a role in early Golgi transport and in recruiting coat components to *trans*-Golgi membranes [55].

Relative to the control, the expression level of nitrosylated sodium/potassium-transporting ATPase subunit beta-1 was increased by ~5-fold in brain exposed to 0.1 Gy, but was unchanged following exposure to 4 Gy. This protein maintains transmembrane electrochemical gradients in all mammalian cells through the transport of extracellular K^+^ in exchange for intracellular Na^+^ [56]. Evidently, modifications that affect this function would result in important physiological perturbations as the concentration gradients of Na^+^ and K^+^ ions across the plasma membrane are essential for maintaining cellular homeostasis [57].

Clearly, investigating transient and persistent radiation-induced alterations in covalent modifications of stress-responsive cellular proteins would enhance our understanding of the health hazards of exposure to low-dose radiation. The availability of specific antibodies to SNO proteins found to be altered by low-and high-dose γ-rays, coupled with *in situ* immune-detection in tissue sections of control and irradiated brains of mice, would be informative of the affected tissues and cells. Together with studies of nitric oxide synthase (neuronal or inducible) activity and measurements of NO production, the studies of the modulation of SNO proteins would enhance our understanding of signaling events in the brain under stress conditions. Notably, long-term studies that involve both physiological and behavioral/motor functions would be a major step toward the understanding of the mechanisms leading to neuronal degeneration. Such studies may contribute to the formulation of strategies to attenuate harmful conditions. Specifically, clinical trials involving treatments with nitric oxide signal-transduction downregulators were found to attenuate neurotoxicity [58]. Our IPA analyses suggest that neuronal nitric oxide synthase (nNOS) signaling is inhibited by low-dose γ-rays and activated by a high dose (Figure 4). Whereas total NOS activity was found to be increased in brain shortly (within 30–60 min) after exposure of pregnant Wistar rats to a 1-Gy dose of γ-rays [59], studies on the regulation of nNOS activation as a function of time following *in vivo* irradiation are lacking. Such studies would be pertinent, since nNOS inhibitors are being investigated as a novel strategy for the therapy and prevention of human melanoma [59], which is often treated with radiation [60]. However, the statistical significance of our results (*p*-value and Z-score) needs to be interpreted cautiously, since the “IPA Knowledge Base” is based on gene/protein expression and not on the amount of nitrosylated protein. Although the identity of canonical pathways and networks involving the uploaded proteins should remain unchanged, statistical significance calculations need to be modified to accommodate the fact that not all proteins contain free cysteine that can be nitrosylated.

There is a long history of research on radiation damage to DNA and proteins [61]. Such damage can be either direct or mediated by radiolysis of water and/or activation of oxidases and nitric oxide synthases that results in the creation of reactive species [2]. The generation of excess levels of reactive oxygen and nitrogen species damages macromolecules, including enzymes [62,63], and perturbs signal transduction processes [64]. Here, we have examined post-translational modifications involving nitrosylation, a topic that has significant relevance to disease development [65]. Notably, both our Western blot and mass spectrometry results suggest that an increase in radiation dose does not necessarily result in increased effects on *S*-nitrosylation. Though the specific nitric oxide synthases are implicated, the interrelated protein clusters that are regulated by radiation-induced *S*-nitrosylation and the link to neurodegenerative conditions remain to be investigated.

## 5. Conclusions

The development of sensitive and quantitative proteomic approaches is highly pertinent to understanding the biological effects of ionizing radiation. Characterizing the *S*-nitrosocysteine proteomes of different organs that vary in their inherent sensitivity to ionizing radiation, together with the application of computational tools to understand functional pathways, will shed further light on the role of structural changes [66] and of the microenvironment in modulating short- and long-term responses to low- and high-dose radiation exposures. This is particularly significant when performed in both male and female animals, exposed to an expanded range of doses delivered in acute single or fractionated boluses or chronically at a low-dose rate. Such studies are relevant to radiation protection and to radiotherapy [67] and may enhance our understanding of the factors that determine radiation sensitivity and the propagation of radiation-induced damaging effects, in particular those contributed by nitric oxide biology [68].

## Supplementary Materials

Supplementary materials can be accessed at: http://www.mdpi.com/2227-7382/03/02/0056/s1.
