# Supplementary material for: S-Nitrosylation in Organs of Mice Exposed to Low or High Doses of γ-Rays: The Modulating Effect of Iodine Contrast Agent at a Low Radiation Dose
Source: Proteomes. 2015 Apr 30;3(2):56–73. doi: 10.3390/proteomes3020056 (PMC4548934; doi:10.3390/proteomes3020056)
Supplement: Supplementary file 1 [file proteomes-03-00056-s001.pdf]

Supplementary Materials

Table 1. Identification of streptavidin enriched biotinylated proteins from brain of 17 week-old C57BL/6J mice exposed to 0, 0.1 or 4 Gy of ^{137}Cs γ-rays 13 days earlier. Equal mass aliquots of protein lysates from 5 different mice in each group were combined, modified by the biotin switch assay, and submitted for mass spectrometry analyses.

Protein Descriptions	Protein Name (Swissprot)	Molecular Weight	Quantitative Value		
			0 cGy	0.1 Gy	4 Gy
14-3-3 protein gamma OS = Mus musculus GN = Ywhag PE = 1 SV = 2	1433G_MOUSE	28 kDa	4.69	7.01	4.64
14-3-3 protein zeta/delta OS = Mus musculus GN = Ywhaz PE = 1 SV = 1	1433Z_MOUSE	28 kDa	14.06	12.85	10.21
1-phosphatidylinositol 4,5-bisphosphate phosphodiesterase beta-1 OS = Mus musculus GN = Plcb1 PE = 1 SV = 2	PLCB1_MOUSE	138 kDa	4.69	4.67	1.86
2',3'-cyclic-nucleotide 3'-phosphodiesterase OS = Mus musculus GN = Cnp PE = 1 SV = 3	CN37_MOUSE	47 kDa	6.56	5.84	11.14
2-oxoglutarate dehydrogenase, mitochondrial OS = Mus musculus GN = Ogdh PE = 1 SV = 3	ODO1_MOUSE	116 kDa	3.75	4.67	4.64
3-hydroxyisobutyrate dehydrogenase, mitochondrial OS = Mus musculus GN = Hibadh PE = 2 SV = 1	3HIDH_MOUSE	35 kDa	0.94	4.67	0.93
3-hydroxyisobutyryl-CoA hydrolase, mitochondrial OS = Mus musculus GN = Hibch PE = 1 SV = 1	HIBCH_MOUSE	43 kDa	0.00	1.17	3.71
3-mercaptopyruvate sulfurtransferase OS = Mus musculus GN = Mpst PE = 1 SV = 3	THTM_MOUSE	33 kDa	5.62	4.67	3.71
40S ribosomal protein S10 OS = Mus musculus GN = Rps10 PE = 1 SV = 1	RS10_MOUSE	19 kDa	6.56	5.84	3.71
40S ribosomal protein S2 OS = Mus musculus GN = Rps2 PE = 1 SV = 3	RS2_MOUSE	31 kDa	2.81	2.34	3.71
40S ribosomal protein S3a OS = Mus musculus GN = Rps3a PE = 1 SV = 3	RS3A_MOUSE	30 kDa	1.87	4.67	0.00
4-aminobutyrate aminotransferase, mitochondrial OS = Mus musculus GN = Abat PE = 1 SV = 1	GABT_MOUSE	56 kDa	13.12	16.35	12.07
4F2 cell-surface antigen heavy chain OS = Mus musculus GN = Slc3a2 PE = 1 SV = 1	4F2_MOUSE	58 kDa	3.75	3.50	2.79
5'-nucleotidase domain-containing protein 3 OS = Mus musculus GN = Nt5dc3 PE = 2 SV = 1	NT5D3_MOUSE	63 kDa	3.75	3.50	3.71
60 kDa heat shock protein, mitochondrial OS = Mus musculus GN = Hspd1 PE = 1 SV = 1	CH60_MOUSE	61 kDa	2.81	1.17	0.00
60S ribosomal protein L5 OS = Mus musculus GN = Rpl5 PE = 1 SV = 3	RL5_MOUSE	34 kDa	3.75	3.50	4.64
6-phosphofructokinase, liver type OS = Mus musculus GN = Pfkl PE = 1 SV = 4	K6PL_MOUSE	85 kDa	3.75	0.00	5.57
6-phosphofructokinase, muscle type OS = Mus musculus GN = Pfkm PE = 1 SV = 3	K6PF_MOUSE	85 kDa	6.56	2.34	7.43
Aconitate hydratase, mitochondrial OS = Mus musculus GN = Aco2 PE = 1 SV = 1	ACON_MOUSE	85 kDa	8.44	5.84	13.00

Table S1. *Cont.*

Protein Descriptions	Protein Name (Swissprot)	Molecular Weight	Quantitative Value		
			0 cGy	0.1 Gy	4 Gy
Actin, cytoplasmic 1 OS = Mus musculus GN = Actb PE = 1 SV = 1	ACTB_MOUSE (+1)	42 kDa	68.43	73.59	56.63
Actin-related protein 2 OS = Mus musculus GN = Actr2 PE = 1 SV = 1	ARP2_MOUSE	45 kDa	17.81	17.52	18.57
Actin-related protein 2/3 complex subunit 2 OS = Mus musculus GN = Arpc2 PE = 1 SV = 3	ARPC2_MOUSE	34 kDa	1.87	9.35	1.86
Actin-related protein 2/3 complex subunit 4 OS = Mus musculus GN = Arpc4 PE = 1 SV = 3	ARPC4_MOUSE	20 kDa	6.56	15.19	9.28
Actin-related protein 3 OS = Mus musculus GN = Actr3 PE = 1 SV = 3	ARP3_MOUSE	47 kDa	12.19	10.51	16.71
Actin-related protein 3B OS = Mus musculus GN = Actr3b PE = 2 SV = 1	ARP3B_MOUSE	48 kDa	10.31	10.51	14.85
Active breakpoint cluster region-related protein OS = Mus musculus GN = Abr PE = 2 SV = 1	ABR_MOUSE	98 kDa	1.87	0.00	2.79
Adapter molecule crk OS = Mus musculus GN = Crk PE = 1 SV = 1	CRK_MOUSE	34 kDa	2.81	4.67	3.71
Adenylate kinase isoenzyme 1 OS = Mus musculus GN = Ak1 PE = 1 SV = 1	KAD1_MOUSE	22 kDa	3.75	1.17	1.86
ADP/ATP translocase 1 OS = Mus musculus GN = Slc25a4 PE = 1 SV = 4	ADT1_MOUSE	33 kDa	30.00	47.89	37.14
ADP/ATP translocase 2 OS = Mus musculus GN = Slc25a5 PE = 1 SV = 3	ADT2_MOUSE	33 kDa	27.18	31.54	24.14
ADP-ribosylation factor 5 OS = Mus musculus GN = Arf5 PE = 2 SV = 2	ARF5_MOUSE	21 kDa	108.74	115.64	150.40
Alanine--tRNA ligase, cytoplasmic OS = Mus musculus GN = Aars PE = 1 SV = 1	SYAC_MOUSE	107 kDa	0.94	2.34	0.93
Aldose reductase OS = Mus musculus GN = Akr1b1 PE = 1 SV = 3	ALDR_MOUSE	36 kDa	4.69	0.00	4.64
Alpha-actinin-4 OS = Mus musculus GN = Actn4 PE = 1 SV = 1	ACTN4_MOUSE	105 kDa	7.50	7.01	3.71
Alpha-adducin OS = Mus musculus GN = Add1 PE = 1 SV = 2	ADDA_MOUSE	81 kDa	3.75	5.84	2.79
Alpha-enolase OS = Mus musculus GN = Eno1 PE = 1 SV = 3	ENOA_MOUSE	47 kDa	51.56	35.04	29.71
Alpha-internexin OS = Mus musculus GN = Ina PE = 1 SV = 2	AINX_MOUSE	56 kDa	3.75	0.00	1.86
Alpha-synuclein OS = Mus musculus GN = Snca PE = 1 SV = 2	SYUA_MOUSE	14 kDa	3.75	0.00	1.86
Ankyrin-2 OS = Mus musculus GN = Ank2 PE = 1 SV = 2	ANK2_MOUSE	426 kDa	1.87	3.50	1.86
Annexin A2 OS = Mus musculus GN = Anxa2 PE = 1 SV = 2	ANXA2_MOUSE	39 kDa	2.81	0.00	0.00

Table S1. *Cont.*

Protein Descriptions	Protein Name (Swissprot)	Molecular Weight	Quantitative Value		
			0 cGy	0.1 Gy	4 Gy
AP-1 complex subunit mu-1 OS = Mus musculus GN = Ap1m1 PE = 1 SV = 3	AP1M1_MOUSE	49 kDa	5.62	0.00	2.79
AP-2 complex subunit alpha-1 OS = Mus musculus GN = Ap2a1 PE = 1 SV = 1	AP2A1_MOUSE	108 kDa	8.44	5.84	13.93
AP-2 complex subunit alpha-2 OS = Mus musculus GN = Ap2a2 PE = 1 SV = 2	AP2A2_MOUSE	104 kDa	8.44	11.68	11.14
AP-2 complex subunit beta OS = Mus musculus GN = Ap2b1 PE = 1 SV = 1	AP2B1_MOUSE	105 kDa	7.50	7.01	13.00
AP-2 complex subunit mu OS = Mus musculus GN = Ap2m1 PE = 1 SV = 1	AP2M1_MOUSE	50 kDa	10.31	7.01	8.36
AP-2 complex subunit sigma OS = Mus musculus GN = Ap2s1 PE = 1 SV = 1	AP2S1_MOUSE	17 kDa	6.56	4.67	5.57
Aspartate aminotransferase, cytoplasmic OS = Mus musculus GN = Got1 PE = 1 SV = 3	AATC_MOUSE	46 kDa	32.81	46.73	27.85
Aspartate aminotransferase, mitochondrial OS = Mus musculus GN = Got2 PE = 1 SV = 1	AATM_MOUSE	47 kDa	12.19	12.85	11.14
Atlastin-1 OS = Mus musculus GN = Atl1 PE = 1 SV = 1	ATLA1_MOUSE	63 kDa	7.50	4.67	5.57
ATP synthase subunit alpha, mitochondrial OS = Mus musculus GN = Atp5a1 PE = 1 SV = 1	ATPA_MOUSE	60 kDa	34.68	49.06	38.99
ATP synthase subunit b, mitochondrial OS = Mus musculus GN = Atp5f1 PE = 1 SV = 1	AT5F1_MOUSE	29 kDa	25.31	33.88	25.07
ATP synthase subunit beta, mitochondrial OS = Mus musculus GN = Atp5b PE = 1 SV = 2	ATPB_MOUSE	56 kDa	31.87	18.69	25.07
ATP synthase subunit d, mitochondrial OS = Mus musculus GN = Atp5h PE = 1 SV = 3	ATP5H_MOUSE	19 kDa	10.31	0.00	12.07
ATP synthase subunit gamma, mitochondrial OS = Mus musculus GN = Atp5c1 PE = 1 SV = 1	ATPG_MOUSE	33 kDa	15.00	7.01	15.78
Band 4.1-like protein 3 OS = Mus musculus GN = Epb41l3 PE = 1 SV = 1	E41L3_MOUSE	103 kDa	5.62	2.34	3.71
Beta-actin-like protein 2 OS = Mus musculus GN = Actbl2 PE = 1 SV = 1	ACTBL_MOUSE	42 kDa	26.25	16.35	23.21
Beta-adducin OS = Mus musculus GN = Add2 PE = 1 SV = 4	ADDB_MOUSE	81 kDa	1.87	0.00	0.93
Beta-adrenergic receptor kinase 1 OS = Mus musculus GN = Adrbk1 PE = 2 SV = 2	ARBK1_MOUSE	80 kDa	1.87	2.34	2.79
Beta-centractin OS = Mus musculus GN = Actr1b PE = 1 SV = 1	ACTY_MOUSE (+1)	42 kDa	5.62	4.67	5.57
Beta-hexosaminidase subunit beta OS = Mus musculus GN = Hexb PE = 2 SV = 2	HEXB_MOUSE	61 kDa	1.87	3.50	2.79
Beta-soluble NSF attachment protein OS = Mus musculus GN = Napb PE = 1 SV = 2	SNAB_MOUSE	34 kDa	5.62	1.17	5.57

Table S1. *Cont.*

Protein Descriptions	Protein Name (Swissprot)	Molecular Weight	Quantitative Value		
			0 cGy	0.1 Gy	4 Gy
Beta-synuclein OS = Mus musculus GN = Sncb PE = 1 SV = 1	SYUB_MOUSE	14 kDa	3.75	5.84	3.71
Brain protein 44 OS = Mus musculus GN = Brp44 PE = 1 SV = 1	BR44_MOUSE	?	6.56	4.67	4.64
Brevican core protein OS = Mus musculus GN = Bcan PE = 1 SV = 2	PGCB_MOUSE	96 kDa	1.87	2.34	3.71
BTB/POZ domain-containing protein 17 OS = Mus musculus GN = Btbd17 PE = 2 SV = 1	BTBDH_MOUSE	53 kDa	2.81	2.34	2.79
BTB/POZ domain-containing protein KCTD16 OS = Mus musculus GN = Kctd16 PE = 1 SV = 2	KCD16_MOUSE	49 kDa	3.75	7.01	3.71
Calcium/calmodulin-dependent 3',5'-cyclic nucleotide phosphodiesterase 1A OS = Mus musculus GN = Pde1a PE = 2 SV = 2	PDE1A_MOUSE (+1)	65 kDa	3.75	2.34	0.00
Calcium/calmodulin-dependent protein kinase type II subunit alpha OS = Mus musculus GN = Camk2a PE = 1 SV = 2	KCC2A_MOUSE	54 kDa	41.25	78.26	58.49
Calcium/calmodulin-dependent protein kinase type II subunit beta OS = Mus musculus GN = Camk2b PE = 1 SV = 2	KCC2B_MOUSE	60 kDa	9.37	7.01	6.50
Calcium/calmodulin-dependent protein kinase type II subunit delta OS = Mus musculus GN = Camk2d PE = 1 SV = 1	KCC2D_MOUSE	56 kDa	6.56	4.67	1.86
Calcium-activated potassium channel subunit alpha-1 OS = Mus musculus GN = Kcnma1 PE = 1 SV = 2	KCMA1_MOUSE	134 kDa	3.75	8.18	6.50
Calcium-binding mitochondrial carrier protein Aralar1 OS = Mus musculus GN = Slc25a12 PE = 1 SV = 1	CMC1_MOUSE	75 kDa	9.37	9.35	11.14
Calcium-dependent secretion activator 1 OS = Mus musculus GN = Cadps PE = 1 SV = 3	CAPS1_MOUSE	153 kDa	11.25	25.70	13.00
Calmodulin OS = Mus musculus GN = Calm1 PE = 1 SV = 2	CALM_MOUSE	17 kDa	1.87	0.00	1.86
Calreticulin OS = Mus musculus GN = Calr PE = 1 SV = 1	CALR_MOUSE	48 kDa	10.31	1.17	6.50
cAMP-dependent protein kinase catalytic subunit alpha OS = Mus musculus GN = Prkaca PE = 1 SV = 3	KAPCA_MOUSE (+1)	41 kDa	3.75	8.18	0.00
Carbonic anhydrase 2 OS = Mus musculus GN = Ca2 PE = 1 SV = 4	CAH2_MOUSE	29 kDa	7.50	7.01	5.57
Casein kinase II subunit alpha' OS = Mus musculus GN = Csnk2a2 PE = 2 SV = 1	CSK22_MOUSE	41 kDa	0.00	3.50	2.79
Cathepsin B OS = Mus musculus GN = Ctsb PE = 1 SV = 2	CATB_MOUSE	37 kDa	6.56	3.50	5.57

Table S1. *Cont.*

Protein Descriptions	Protein Name (Swissprot)	Molecular Weight	Quantitative Value		
			0 cGy	0.1 Gy	4 Gy
Cell adhesion molecule 3 OS = Mus musculus GN = Cadm3 PE = 1 SV = 1	CADM3_MOUSE	43 kDa	1.87	0.00	1.86
Cell division control protein 42 homolog OS = Mus musculus GN = Cdc42 PE = 1 SV = 2	CDC42_MOUSE	21 kDa	3.75	2.34	3.71
Citrate synthase, mitochondrial OS = Mus musculus GN = Cs PE = 1 SV = 1	CISY_MOUSE	52 kDa	17.81	36.21	21.35
Clathrin coat assembly protein AP180 OS = Mus musculus GN = Snap91 PE = 1 SV = 1	AP180_MOUSE	92 kDa	15.94	7.01	9.28
Clathrin heavy chain 1 OS = Mus musculus GN = Cltc PE = 1 SV = 3	CLH_MOUSE	?	54.37	74.76	69.63
Cofilin-1 OS = Mus musculus GN = Cfl1 PE = 1 SV = 3	COF1_MOUSE	19 kDa	11.25	9.35	10.21
Cofilin-2 OS = Mus musculus GN = Cfl2 PE = 1 SV = 1	COF2_MOUSE	19 kDa	4.69	4.67	3.71
Contactin-1 OS = Mus musculus GN = Cntn1 PE = 1 SV = 1	CNTN1_MOUSE	113 kDa	4.69	3.50	6.50
Contactin-associated protein 1 OS = Mus musculus GN = Cntnap1 PE = 1 SV = 2	CNTP1_MOUSE	156 kDa	0.94	0.00	3.71
Contactin-associated protein-like 2 OS = Mus musculus GN = Cntnap2 PE = 1 SV = 2	CNTP2_MOUSE	148 kDa	5.62	5.84	5.57
Coronin-1C OS = Mus musculus GN = Coro1c PE = 1 SV = 2	COR1C_MOUSE	53 kDa	1.87	0.00	3.71
Creatine kinase B-type OS = Mus musculus GN = Ckb PE = 1 SV = 1	KCRB_MOUSE	43 kDa	37.50	78.26	36.21
Creatine kinase U-type, mitochondrial OS = Mus musculus GN = Ckmt1 PE = 1 SV = 1	KCRU_MOUSE	47 kDa	30.00	18.69	39.92
C-terminal-binding protein 1 OS = Mus musculus GN = Ctbp1 PE = 1 SV = 2	CTBP1_MOUSE	48 kDa	0.00	0.00	2.79
Cullin-associated NEDD8-dissociated protein 1 OS = Mus musculus GN = Cand1 PE = 2 SV = 2	CAND1_MOUSE	136 kDa	0.94	4.67	1.86
Cytochrome b-c1 complex subunit 1, mitochondrial OS = Mus musculus GN = Uqcrc1 PE = 1 SV = 2	QCR1_MOUSE	53 kDa	12.19	14.02	17.64
Cytochrome b-c1 complex subunit 2, mitochondrial OS = Mus musculus GN = Uqcrc2 PE = 1 SV = 1	QCR2_MOUSE	48 kDa	0.00	2.34	1.86
Cytochrome c oxidase subunit 2 OS = Mus musculus GN = Mtco2 PE = 1 SV = 1	COX2_MOUSE	26 kDa	1.87	4.67	3.71
Cytochrome c oxidase subunit 4 isoform 1, mitochondrial OS = Mus musculus GN = Cox4i1 PE = 1 SV = 2	COX41_MOUSE	20 kDa	1.87	0.00	3.71
Cytochrome c oxidase subunit 6B1 OS = Mus musculus GN = Cox6b1 PE = 1 SV = 2	CX6B1_MOUSE	10 kDa	8.44	14.02	10.21
Cytochrome c1, heme protein, mitochondrial OS = Mus musculus GN = Cyc1 PE = 1 SV = 1	CY1_MOUSE	35 kDa	10.31	10.51	9.28
Cytoplasmic dynein 1 heavy chain 1 OS = Mus musculus GN = Dync1h1 PE = 1 SV = 2	DYHC1_MOUSE	532 kDa	2.81	11.68	11.14

Table S1. *Cont.*

Protein Descriptions	Protein Name (Swissprot)	Molecular Weight	Quantitative Value		
			0 cGy	0.1 Gy	4 Gy
Cytoplasmic dynein 1 light intermediate chain 2 OS = Mus musculus GN = Dync1li2 PE = 1 SV = 2	DC1L2_MOUSE	54 kDa	1.87	5.84	3.71
Cytoplasmic FMR1-interacting protein 1 OS = Mus musculus GN = Cyfip1 PE = 1 SV = 1	CYFP1_MOUSE	145 kDa	1.87	0.00	2.79
Cytoplasmic FMR1-interacting protein 2 OS = Mus musculus GN = Cyfip2 PE = 1 SV = 2	CYFP2_MOUSE	146 kDa	0.00	0.00	5.57
Cytosol aminopeptidase OS = Mus musculus GN = Lap3 PE = 1 SV = 3	AMPL_MOUSE	56 kDa	2.81	4.67	3.71
Cytosolic 10-formyltetrahydrofolate dehydrogenase OS = Mus musculus GN = Aldh1l1 PE = 2 SV = 1	AL1L1_MOUSE	99 kDa	5.62	4.67	5.57
D-beta-hydroxybutyrate dehydrogenase, mitochondrial OS = Mus musculus GN = Bdh1 PE = 1 SV = 2	BDH_MOUSE	38 kDa	2.81	5.84	5.57
D-dopachrome decarboxylase OS = Mus musculus GN = Ddt PE = 1 SV = 3	DOPD_MOUSE	13 kDa	1.87	1.17	3.71
Dihydrolipoyllysine-residue acetyltransferase component of pyruvate dehydrogenase complex, mitochondrial OS = Mus musculus GN = Dlat PE = 1 SV = 2	ODP2_MOUSE	68 kDa	1.87	0.00	0.00
Dihydropyrimidinase-related protein 1 OS = Mus musculus GN = Crmp1 PE = 1 SV = 1	DPYL1_MOUSE	62 kDa	12.19	7.01	13.00
Dihydropyrimidinase-related protein 2 OS = Mus musculus GN = Dpysl2 PE = 1 SV = 2	DPYL2_MOUSE	62 kDa	27.18	33.88	28.78
Dihydropyrimidinase-related protein 4 OS = Mus musculus GN = Dpysl4 PE = 1 SV = 1	DPYL4_MOUSE	62 kDa	3.75	2.34	1.86
Dipeptidyl aminopeptidase-like protein 6 OS = Mus musculus GN = Dpp6 PE = 1 SV = 1	DPP6_MOUSE	91 kDa	4.69	2.34	5.57
Disintegrin and metalloproteinase domain-containing protein 22 OS = Mus musculus GN = Adam22 PE = 1 SV = 2	ADA22_MOUSE	100 kDa	0.00	1.17	2.79
Disks large homolog 2 OS = Mus musculus GN = Dlg2 PE = 1 SV = 2	DLG2_MOUSE	95 kDa	5.62	8.18	7.43
Disks large homolog 4 OS = Mus musculus GN = Dlg4 PE = 1 SV = 1	DLG4_MOUSE	80 kDa	5.62	4.67	9.28
DmX-like protein 2 OS = Mus musculus GN = Dmxl2 PE = 1 SV = 3	DMXL2_MOUSE	338 kDa	8.44	11.68	12.07
DOMON domain-containing protein FRRS1L OS = Mus musculus GN = Frrs1l PE = 1 SV = 1	FRS1L_MOUSE	33 kDa	2.81	0.00	0.00
Double C2-like domain-containing protein alpha OS = Mus musculus GN = Doc2a PE = 2 SV = 1	DOC2A_MOUSE	45 kDa	1.87	5.84	1.86
Dynamin-1 OS = Mus musculus GN = Dnm1 PE = 1 SV = 2	DYN1_MOUSE	98 kDa	25.31	29.20	29.71
Dynamin-1-like protein OS = Mus musculus GN = Dnm1l PE = 1 SV = 2	DNM1L_MOUSE	83 kDa	1.87	2.34	2.79

Table S1. *Cont.*

Protein Descriptions	Protein Name (Swissprot)	Molecular Weight	Quantitative Value		
			0 cGy	0.1 Gy	4 Gy
Dynamin-like 120 kDa protein, mitochondrial OS = Mus musculus GN = Opa1 PE = 1 SV = 1	OPA1_MOUSE	111 kDa	2.81	5.84	2.79
E3 ubiquitin-protein ligase UBR4 OS = Mus musculus GN = Ubr4 PE = 1 SV = 1	UBR4_MOUSE	572 kDa	1.87	0.00	2.79
EH domain-containing protein 1 OS = Mus musculus GN = Ehd1 PE = 1 SV = 1	EHD1_MOUSE (+1)	61 kDa	2.81	7.01	5.57
Elongation factor 1-alpha 1 OS = Mus musculus GN = Eef1a1 PE = 1 SV = 3	EF1A1_MOUSE	50 kDa	15.00	11.68	15.78
Elongation factor 1-alpha 2 OS = Mus musculus GN = Eef1a2 PE = 1 SV = 1	EF1A2_MOUSE	50 kDa	12.19	11.68	19.50
Elongation factor 1-gamma OS = Mus musculus GN = Eef1g PE = 1 SV = 3	EF1G_MOUSE	50 kDa	6.56	2.34	3.71
Elongation factor 2 OS = Mus musculus GN = Eef2 PE = 1 SV = 2	EF2_MOUSE	95 kDa	4.69	10.51	1.86
Endophilin-B2 OS = Mus musculus GN = Sh3glb2 PE = 2 SV = 2	SHLB2_MOUSE	45 kDa	1.87	0.00	3.71
Endoplasmin OS = Mus musculus GN = Hsp90b1 PE = 1 SV = 2	ENPL_MOUSE	92 kDa	3.75	2.34	2.79
Eukaryotic peptide chain release factor GTP-binding subunit ERF3A OS = Mus musculus GN = Gspt1 PE = 1 SV = 2	ERF3A_MOUSE	69 kDa	4.69	4.67	3.71
Eukaryotic translation initiation factor 5A-1 OS = Mus musculus GN = Eif5a PE = 1 SV = 2	IF5A1_MOUSE	17 kDa	4.69	1.17	0.00
Excitatory amino acid transporter 1 OS = Mus musculus GN = Slc1a3 PE = 1 SV = 2	EAA1_MOUSE	60 kDa	1.87	2.34	5.57
Excitatory amino acid transporter 2 OS = Mus musculus GN = Slc1a2 PE = 1 SV = 1	EAA2_MOUSE	62 kDa	8.44	2.34	10.21
F-actin-capping protein subunit beta OS = Mus musculus GN = Capzb PE = 1 SV = 3	CAPZB_MOUSE	31 kDa	5.62	0.00	5.57
Fascin OS = Mus musculus GN = Fscn1 PE = 1 SV = 4	FSCN1_MOUSE	55 kDa	0.00	0.00	2.79
Fatty acid synthase OS = Mus musculus GN = Fasn PE = 1 SV = 2	FAS_MOUSE	272 kDa	1.87	3.50	3.71
Fatty acid-binding protein, heart OS = Mus musculus GN = Fabp3 PE = 1 SV = 5	FABPH_MOUSE	15 kDa	4.69	0.00	4.64
F-box/LRR-repeat protein 16 OS = Mus musculus GN = Fbxl16 PE = 2 SV = 1	FXL16_MOUSE	52 kDa	0.00	0.00	3.71
Formin-binding protein 1-like OS = Mus musculus GN = Fnbp1l PE = 1 SV = 2	FBP1L_MOUSE	70 kDa	2.81	2.34	3.71
Fructose-bisphosphate aldolase A OS = Mus musculus GN = Aldoa PE = 1 SV = 2	ALDOA_MOUSE	39 kDa	4.69	3.50	5.57
Fumarate hydratase, mitochondrial OS = Mus musculus GN = Fh PE = 1 SV = 3	FUMH_MOUSE	54 kDa	0.00	3.50	0.00

Table S1. *Cont.*

Protein Descriptions	Protein Name (Swissprot)	Molecular Weight	Quantitative Value		
			0 cGy	0.1 Gy	4 Gy
Gamma-enolase OS = Mus musculus GN = Eno2 PE = 1 SV = 2	ENOG_MOUSE	47 kDa	64.68	31.54	31.57
Glucose-6-phosphate 1-dehydrogenase X OS = Mus musculus GN = G6pdx PE = 1 SV = 3	G6PD1_MOUSE	59 kDa	3.75	2.34	5.57
Glucose-6-phosphate isomerase OS = Mus musculus GN = Gpi PE = 1 SV = 4	G6PI_MOUSE	63 kDa	7.50	7.01	4.64
Glucosidase 2 subunit beta OS = Mus musculus GN = Prkcsh PE = 1 SV = 1	GLU2B_MOUSE	59 kDa	3.75	4.67	3.71
Glutamate decarboxylase 2 OS = Mus musculus GN = Gad2 PE = 2 SV = 1	DCE2_MOUSE	65 kDa	3.75	0.00	4.64
Glutamate dehydrogenase 1, mitochondrial OS = Mus musculus GN = Glud1 PE = 1 SV = 1	DHE3_MOUSE	61 kDa	6.56	4.67	7.43
Glutamate receptor ionotropic, NMDA 1 OS = Mus musculus GN = Grin1 PE = 1 SV = 1	NMDZ1_MOUSE	105 kDa	1.87	0.00	2.79
Glutamate receptor ionotropic, NMDA 2B OS = Mus musculus GN = Grin2b PE = 1 SV = 3	NMDE2_MOUSE	166 kDa	1.87	1.17	3.71
Glutaminase kidney isoform, mitochondrial OS = Mus musculus GN = Gls PE = 1 SV = 1	GLSK_MOUSE	74 kDa	3.75	5.84	5.57
Glutamine synthetase OS = Mus musculus GN = Glul PE = 1 SV = 6	GLNA_MOUSE	42 kDa	19.69	11.68	22.28
Glutaredoxin-3 OS = Mus musculus GN = Glrx3 PE = 1 SV = 1	GLRX3_MOUSE	38 kDa	3.75	2.34	3.71
Glutathione S-transferase Mu 1 OS = Mus musculus GN = Gstm1 PE = 1 SV = 2	GSTM1_MOUSE	26 kDa	3.75	8.18	3.71
Glutathione S-transferase Mu 5 OS = Mus musculus GN = Gstm5 PE = 1 SV = 1	GSTM5_MOUSE	27 kDa	5.62	2.34	5.57
Glyceraldehyde-3-phosphate dehydrogenase OS = Mus musculus GN = Gapdh PE = 1 SV = 2	G3P_MOUSE	36 kDa	20.62	17.52	24.14
Glycerol-3-phosphate dehydrogenase, mitochondrial OS = Mus musculus GN = Gpd2 PE = 1 SV = 2	GPDM_MOUSE	81 kDa	3.75	5.84	4.64
Glycogen phosphorylase, brain form OS = Mus musculus GN = Pygb PE = 1 SV = 3	PYGB_MOUSE	97 kDa	6.56	9.35	9.28
Glyoxalase domain-containing protein 4 OS = Mus musculus GN = Glod4 PE = 2 SV = 1	GLOD4_MOUSE	33 kDa	3.75	5.84	4.64
GMP synthase [glutamine-hydrolyzing] OS = Mus musculus GN = Gmps PE = 1 SV = 2	GUAA_MOUSE	77 kDa	3.75	1.17	1.86
GTP:AMP phosphotransferase AK3, mitochondrial OS = Mus musculus GN = Ak3 PE = 1 SV = 3	KAD3_MOUSE	25 kDa	1.87	4.67	1.86
GTP-binding nuclear protein Ran OS = Mus musculus GN = Ran PE = 1 SV = 3	RAN_MOUSE	24 kDa	3.75	7.01	4.64
Guanine deaminase OS = Mus musculus GN = Gda PE = 1 SV = 1	GUAD_MOUSE	51 kDa	1.87	3.50	1.86

Table S1. *Cont.*

Protein Descriptions	Protein Name (Swissprot)	Molecular Weight	Quantitative Value		
			0 cGy	0.1 Gy	4 Gy
Guanine nucleotide-binding protein G(i) subunit alpha-1 OS = Mus musculus GN = Gnai1 PE = 2 SV = 1	GNAI1_MOUSE	40 kDa	9.37	7.01	7.43
Guanine nucleotide-binding protein G(I)/G(S)/G(T) subunit beta-1 OS = Mus musculus GN = Gnb1 PE = 1 SV = 3	GBB1_MOUSE	37 kDa	19.69	17.52	11.14
Guanine nucleotide-binding protein G(I)/G(S)/G(T) subunit beta-2 OS = Mus musculus GN = Gnb2 PE = 1 SV = 3	GBB2_MOUSE	37 kDa	17.81	17.52	7.43
Guanine nucleotide-binding protein G(o) subunit alpha OS = Mus musculus GN = Gnao1 PE = 1 SV = 3	GNAO_MOUSE	40 kDa	9.37	5.84	13.00
Guanine nucleotide-binding protein G(q) subunit alpha OS = Mus musculus GN = Gnaq PE = 1 SV = 4	GNAQ_MOUSE	42 kDa	1.87	8.18	0.00
Heat shock 70 kDa protein 12A OS = Mus musculus GN = Hspa12a PE = 1 SV = 1	HS12A_MOUSE	75 kDa	2.81	4.67	1.86
Heat shock cognate 71 kDa protein OS = Mus musculus GN = Hspa8 PE = 1 SV = 1	HSP7C_MOUSE	71 kDa	48.74	66.58	72.41
Heat shock protein HSP 90-alpha OS = Mus musculus GN = Hsp90aa1 PE = 1 SV = 4	HS90A_MOUSE	85 kDa	63.74	77.10	75.20
Heat shock protein HSP 90-beta OS = Mus musculus GN = Hsp90ab1 PE = 1 SV = 3	HS90B_MOUSE	83 kDa	47.81	53.73	54.78
Hemoglobin subunit alpha OS = Mus musculus GN = Hba PE = 1 SV = 2	HBA_MOUSE	15 kDa	20.62	24.53	15.78
Hemoglobin subunit beta-1 OS = Mus musculus GN = Hbb-b1 PE = 1 SV = 2	HBB1_MOUSE	16 kDa	33.75	23.36	23.21
Heterogeneous nuclear ribonucleoprotein A/B OS = Mus musculus GN = Hnrnpab PE = 1 SV = 1	ROAA_MOUSE	31 kDa	4.69	0.00	5.57
Heterogeneous nuclear ribonucleoprotein A1 OS = Mus musculus GN = Hnrnpa1 PE = 1 SV = 2	ROA1_MOUSE	34 kDa	3.75	3.50	4.64
Heterogeneous nuclear ribonucleoprotein A3 OS = Mus musculus GN = Hnrnpa3 PE = 1 SV = 1	ROA3_MOUSE	40 kDa	2.81	0.00	3.71
Heterogeneous nuclear ribonucleoprotein D0 OS = Mus musculus GN = Hnrnpd PE = 1 SV = 2	HNRPD_MOUSE	38 kDa	3.75	0.00	5.57
Heterogeneous nuclear ribonucleoprotein U OS = Mus musculus GN = Hnrnpu PE = 1 SV = 1	HNRPU_MOUSE	88 kDa	5.62	0.00	1.86
Heterogeneous nuclear ribonucleoproteins A2/B1 OS = Mus musculus GN = Hnrnpa2b1 PE = 1 SV = 2	ROA2_MOUSE	37 kDa	1.87	1.17	3.71
Hexokinase-1 OS = Mus musculus GN = Hk1 PE = 1 SV = 3	HXK1_MOUSE	108 kDa	12.19	12.85	15.78
Histidine triad nucleotide-binding protein 1 OS = Mus musculus GN = Hint1 PE = 1 SV = 3	HINT1_MOUSE	14 kDa	5.62	16.35	11.14

Table S1. *Cont.*

Protein Descriptions	Protein Name (Swissprot)	Molecular Weight	Quantitative Value		
			0 cGy	0.1 Gy	4 Gy
Histone H2A type 2-A OS = Mus musculus GN = Hist2h2aa1 PE = 1 SV = 3	H2A2A_MOUSE (+1)	14 kDa	10.31	2.34	5.57
Histone H4 OS = Mus musculus GN = Hist1h4a PE = 1 SV = 2	H4_MOUSE	11 kDa	8.44	2.34	5.57
Homer protein homolog 1 OS = Mus musculus GN = Homer1 PE = 1 SV = 2	HOME1_MOUSE	41 kDa	5.62	7.01	7.43
Hyaluronan and proteoglycan link protein 1 OS = Mus musculus GN = Hapln1 PE = 2 SV = 1	HPLN1_MOUSE	40 kDa	3.75	2.34	2.79
Hypoxanthine-guanine phosphoribosyltransferase OS = Mus musculus GN = Hprt1 PE = 1 SV = 3	HPRT_MOUSE	25 kDa	2.81	1.17	3.71
Hypoxia up-regulated protein 1 OS = Mus musculus GN = Hyou1 PE = 1 SV = 1	HYOU1_MOUSE	111 kDa	3.75	4.67	3.71
Immunoglobulin superfamily member 8 OS = Mus musculus GN = Igsf8 PE = 1 SV = 2	IGSF8_MOUSE	65 kDa	6.56	0.00	4.64
Inactive dipeptidyl peptidase 10 OS = Mus musculus GN = Dpp10 PE = 2 SV = 1	DPP10_MOUSE	91 kDa	2.81	3.50	1.86
Inositol 1,4,5-trisphosphate receptor type 1 OS = Mus musculus GN = Itpr1 PE = 1 SV = 2	ITPR1_MOUSE	313 kDa	1.87	8.18	4.64
Isocitrate dehydrogenase [NAD] subunit alpha, mitochondrial OS = Mus musculus GN = Idh3a PE = 1 SV = 1	IDH3A_MOUSE	40 kDa	4.69	2.34	6.50
Isocitrate dehydrogenase [NAD] subunit gamma 1, mitochondrial OS = Mus musculus GN = Idh3g PE = 1 SV = 1	IDHG1_MOUSE	43 kDa	7.50	15.19	7.43
Isoleucine--tRNA ligase, mitochondrial OS = Mus musculus GN = Iars2 PE = 2 SV = 1	SYIM_MOUSE	113 kDa	3.75	1.17	0.93
Kinesin light chain 1 OS = Mus musculus GN = Klc1 PE = 1 SV = 3	KLC1_MOUSE	61 kDa	1.87	0.00	3.71
Lactoylglutathione lyase OS = Mus musculus GN = Glo1 PE = 1 SV = 3	LGUL_MOUSE	21 kDa	1.87	0.00	0.93
Leukotriene A-4 hydrolase OS = Mus musculus GN = Lta4h PE = 1 SV = 4	LKHA4_MOUSE	69 kDa	1.87	3.50	2.79
Limbic system-associated membrane protein OS = Mus musculus GN = Lsamp PE = 1 SV = 1	LSAMP_MOUSE	38 kDa	2.81	0.00	1.86
L-lactate dehydrogenase B chain OS = Mus musculus GN = Ldhb PE = 1 SV = 2	LDHB_MOUSE	37 kDa	8.44	2.34	5.57
Long-chain-fatty-acid--CoA ligase ACSBG1 OS = Mus musculus GN = Acsbg1 PE = 1 SV = 1	ACBG1_MOUSE	80 kDa	1.87	2.34	2.79
Malate dehydrogenase, cytoplasmic OS = Mus musculus GN = Mdh1 PE = 1 SV = 3	MDHC_MOUSE	37 kDa	8.44	4.67	6.50
Malate dehydrogenase, mitochondrial OS = Mus musculus GN = Mdh2 PE = 1 SV = 3	MDHM_MOUSE	36 kDa	7.50	2.34	9.28

Table S1. *Cont.*

Protein Descriptions	Protein Name (Swissprot)	Molecular Weight	Quantitative Value		
			0 cGy	0.1 Gy	4 Gy
MAP kinase-activating death domain protein OS = Mus musculus GN = Madd PE = 1 SV = 2	MADD_MOUSE	175 kDa	1.87	2.34	3.71
Matrin-3 OS = Mus musculus GN = Matr3 PE = 1 SV = 1	MATR3_MOUSE	95 kDa	1.87	4.67	1.86
Membrane-associated guanylate kinase, WW and PDZ domain-containing protein 2 OS = Mus musculus GN = Magi2 PE = 1 SV = 2	MAGI2_MOUSE	141 kDa	3.75	7.01	5.57
Methylcrotonoyl-CoA carboxylase subunit alpha, mitochondrial OS = Mus musculus GN = Mccc1 PE = 2 SV = 2	MCCA_MOUSE	79 kDa	1.87	9.35	5.57
Microsomal glutathione S-transferase 3 OS = Mus musculus GN = Mgst3 PE = 1 SV = 1	MGST3_MOUSE	17 kDa	2.81	2.34	2.79
Microtubule-associated protein 2 OS = Mus musculus GN = Map2 PE = 1 SV = 2	MAP2_MOUSE	199 kDa	0.00	0.00	2.79
Microtubule-associated protein RP/EB family member 3 OS = Mus musculus GN = Mapre3 PE = 1 SV = 1	MARE3_MOUSE	32 kDa	1.87	4.67	2.79
Microtubule-associated protein tau OS = Mus musculus GN = Mapt PE = 1 SV = 3	TAU_MOUSE	76 kDa	9.37	3.50	5.57
Mitochondrial 2-oxoglutarate/malate carrier protein OS = Mus musculus GN = Slc25a11 PE = 1 SV = 3	M2OM_MOUSE	34 kDa	4.69	11.68	7.43
Mitochondrial carrier homolog 2 OS = Mus musculus GN = Mtch2 PE = 1 SV = 1	MTCH2_MOUSE	33 kDa	2.81	2.34	3.71
Mitochondrial glutamate carrier 1 OS = Mus musculus GN = Slc25a22 PE = 1 SV = 1	GHC1_MOUSE	35 kDa	4.69	0.00	3.71
Mitochondrial import receptor subunit TOM70 OS = Mus musculus GN = Tomm70a PE = 1 SV = 2	TOM70_MOUSE	68 kDa	3.75	0.00	3.71
Mitochondrial inner membrane protein OS = Mus musculus GN = Immt PE = 1 SV = 1	IMMT_MOUSE	84 kDa	3.75	2.34	2.79
Monoglyceride lipase OS = Mus musculus GN = Mgll PE = 1 SV = 1	MGLL_MOUSE	33 kDa	1.87	2.34	3.71
MOSC domain-containing protein 2, mitochondrial OS = Mus musculus GN = Marc2 PE = 1 SV = 1	MOSC2_MOUSE	38 kDa	1.87	0.00	1.86
Murinoglobulin-1 OS = Mus musculus GN = Mug1 PE = 1 SV = 3	MUG1_MOUSE	165 kDa	5.62	2.34	4.64
Myelin basic protein OS = Mus musculus GN = Mbp PE = 1 SV = 2	MBP_MOUSE	27 kDa	4.69	2.34	7.43
Myelin proteolipid protein OS = Mus musculus GN = Plp1 PE = 1 SV = 2	MYPR_MOUSE	30 kDa	24.37	10.51	35.28
Myelin-associated glycoprotein OS = Mus musculus GN = Mag PE = 1 SV = 2	MAG_MOUSE	69 kDa	1.87	3.50	1.86
Myoglobin OS = Mus musculus GN = Mb PE = 1 SV = 3	MYG_MOUSE	17 kDa	2.81	4.67	3.71

Table S1. *Cont.*

Protein Descriptions	Protein Name (Swissprot)	Molecular Weight	Quantitative Value		
			0 cGy	0.1 Gy	4 Gy
Myosin-10 OS = Mus musculus GN = Myh10 PE = 1 SV = 2	MYH10_MOUSE	229 kDa	3.75	2.34	2.79
Myosin-9 OS = Mus musculus GN = Myh9 PE = 1 SV = 4	MYH9_MOUSE	226 kDa	8.44	4.67	3.71
NADH dehydrogenase [ubiquinone] 1 alpha subcomplex subunit 10, mitochondrial OS = Mus musculus GN = Ndufa10 PE = 1 SV = 1	NDUAA_MOUSE	41 kDa	1.87	3.50	4.64
NADH dehydrogenase [ubiquinone] 1 alpha subcomplex subunit 12 OS = Mus musculus GN = Ndufa12 PE = 1 SV = 2	NDUAC_MOUSE	17 kDa	2.81	0.00	1.86
NADH dehydrogenase [ubiquinone] 1 alpha subcomplex subunit 8 OS = Mus musculus GN = Ndufa8 PE = 1 SV = 3	NDUA8_MOUSE	20 kDa	3.75	5.84	4.64
NADH dehydrogenase [ubiquinone] 1 alpha subcomplex subunit 9, mitochondrial OS = Mus musculus GN = Ndufa9 PE = 1 SV = 2	NDUA9_MOUSE	43 kDa	13.12	19.86	15.78
NADH dehydrogenase [ubiquinone] 1 beta subcomplex subunit 9 OS = Mus musculus GN = Ndufb9 PE = 1 SV = 3	NDUB9_MOUSE	22 kDa	4.69	9.35	6.50
NADH dehydrogenase [ubiquinone] flavoprotein 1, mitochondrial OS = Mus musculus GN = Ndufv1 PE = 1 SV = 1	NDUV1_MOUSE	51 kDa	6.56	3.50	7.43
NADH dehydrogenase [ubiquinone] iron-sulfur protein 6, mitochondrial OS = Mus musculus GN = Ndufs6 PE = 1 SV = 2	NDUS6_MOUSE	13 kDa	1.87	0.00	3.71
NADP-dependent malic enzyme, mitochondrial OS = Mus musculus GN = Me3 PE = 1 SV = 2	MAON_MOUSE	67 kDa	5.62	0.00	5.57
Neural cell adhesion molecule 1 OS = Mus musculus GN = Ncam1 PE = 1 SV = 3	NCAM1_MOUSE	119 kDa	4.69	1.17	11.14
Neurobeachin OS = Mus musculus GN = Nbea PE = 1 SV = 1	NBEA_MOUSE	327 kDa	0.00	2.34	4.64
Neurochondrin OS = Mus musculus GN = Ncdn PE = 1 SV = 1	NCDN_MOUSE	79 kDa	2.81	2.34	7.43
Neurofilament light polypeptide OS = Mus musculus GN = Nefl PE = 1 SV = 5	NFL_MOUSE	62 kDa	1.87	0.00	3.71
Neuronal membrane glycoprotein M6-a OS = Mus musculus GN = Gpm6a PE = 1 SV = 1	GPM6A_MOUSE	31 kDa	5.62	1.17	5.57
Neuron-specific calcium-binding protein hippocalcin OS = Mus musculus GN = Hpca PE = 1 SV = 2	HPCA_MOUSE	22 kDa	5.62	2.34	4.64
Neuroplastin OS = Mus musculus GN = Nptn PE = 1 SV = 3	NPTN_MOUSE	44 kDa	2.81	4.67	6.50
Neutral alpha-glucosidase AB OS = Mus musculus GN = Ganab PE = 1 SV = 1	GANAB_MOUSE	107 kDa	0.00	5.84	1.86

Table S1. *Cont.*

Protein Descriptions	Protein Name (Swissprot)	Molecular Weight	Quantitative Value		
			0 cGy	0.1 Gy	4 Gy
Nocturnin OS = Mus musculus GN = Ccrn4l PE = 2 SV = 3	NOCT_MOUSE	48 kDa	3.75	1.17	0.00
Nucleoside diphosphate kinase A OS = Mus musculus GN = Nme1 PE = 1 SV = 1	NDKA_MOUSE	17 kDa	1.87	2.34	4.64
OCIA domain-containing protein 2 OS = Mus musculus GN = Ociad2 PE = 2 SV = 1	OCAD2_MOUSE	17 kDa	3.75	4.67	3.71
Olfactory marker protein OS = Mus musculus GN = Omp PE = 1 SV = 3	OMP_MOUSE	19 kDa	0.00	0.00	3.71
Oxidation resistance protein 1 OS = Mus musculus GN = Oxr1 PE = 1 SV = 3	OXR1_MOUSE	96 kDa	1.87	7.01	7.43
Peptidyl-prolyl cis-trans isomerase A OS = Mus musculus GN = Ppia PE = 1 SV = 2	PPIA_MOUSE	18 kDa	35.62	14.02	30.64
Peptidyl-prolyl cis-trans isomerase B OS = Mus musculus GN = Ppib PE = 2 SV = 2	PPIB_MOUSE	24 kDa	13.12	7.01	13.00
Peptidyl-prolyl cis-trans isomerase FKBP4 OS = Mus musculus GN = Fkbp4 PE = 1 SV = 5	FKBP4_MOUSE	52 kDa	0.94	2.34	2.79
Peptidyl-prolyl cis-trans isomerase NIMA-interacting 1 OS = Mus musculus GN = Pin1 PE = 1 SV = 1	PIN1_MOUSE	18 kDa	2.81	2.34	3.71
Peroxiredoxin-5, mitochondrial OS = Mus musculus GN = Prdx5 PE = 1 SV = 2	PRDX5_MOUSE	22 kDa	3.75	0.00	3.71
Peroxiredoxin-6 OS = Mus musculus GN = Prdx6 PE = 1 SV = 3	PRDX6_MOUSE	25 kDa	3.75	2.34	3.71
Phosphate carrier protein, mitochondrial OS = Mus musculus GN = Slc25a3 PE = 1 SV = 1	MPCP_MOUSE	40 kDa	5.62	2.34	4.64
Phosphatidylethanolamine-binding protein 1 OS = Mus musculus GN = Pebp1 PE = 1 SV = 3	PEBP1_MOUSE	21 kDa	6.56	1.17	2.79
Phosphatidylinositol 3,4,5-trisphosphate 3-phosphatase and dual-specificity protein phosphatase PTEN OS = Mus musculus GN = Pten PE = 1 SV = 1	PTEN_MOUSE	47 kDa	1.87	2.34	3.71
Phosphatidylinositol 4-phosphate 5-kinase type-1 gamma OS = Mus musculus GN = Pip5k1c PE = 1 SV = 2	PI51C_MOUSE	72 kDa	3.75	0.00	3.71
Phosphatidylinositol transfer protein alpha isoform OS = Mus musculus GN = Pitpna PE = 1 SV = 2	PIPNA_MOUSE	32 kDa	2.81	3.50	3.71
Phosphofurin acidic cluster sorting protein 1 OS = Mus musculus GN = Pacs1 PE = 1 SV = 2	PACS1_MOUSE	105 kDa	3.75	4.67	6.50
Phosphoglycerate kinase 1 OS = Mus musculus GN = Pgk1 PE = 1 SV = 4	PGK1_MOUSE	45 kDa	3.75	5.84	5.57
Phosphoglycerate mutase 1 OS = Mus musculus GN = Pgam1 PE = 1 SV = 3	PGAM1_MOUSE	29 kDa	9.37	7.01	9.28
PITH domain-containing protein 1 OS = Mus musculus GN = Pithd1 PE = 2 SV = 1	PITH1_MOUSE	24 kDa	3.75	1.17	3.71

Table S1. *Cont.*

Protein Descriptions	Protein Name (Swissprot)	Molecular Weight	Quantitative Value		
			0 cGy	0.1 Gy	4 Gy
Plasma membrane calcium-transporting ATPase 2 OS = Mus musculus GN = Atp2b2 PE = 1 SV = 2	AT2B2_MOUSE	133 kDa	3.75	10.51	5.57
Plectin OS = Mus musculus GN = Plec PE = 1 SV = 2	PLEC_MOUSE	534 kDa	6.56	4.67	7.43
Probable phospholipid-transporting ATPase IA OS = Mus musculus GN = Atp8a1 PE = 1 SV = 1	AT8A1_MOUSE	130 kDa	8.44	8.18	7.43
Probable ubiquitin carboxyl-terminal hydrolase FAF-X OS = Mus musculus GN = Usp9x PE = 1 SV = 2	USP9X_MOUSE	291 kDa	6.56	3.50	7.43
Profilin-1 OS = Mus musculus GN = Pfn1 PE = 1 SV = 2	PROF1_MOUSE	15 kDa	5.62	0.00	5.57
Prohibitin OS = Mus musculus GN = Phb PE = 1 SV = 1	PHB_MOUSE	30 kDa	2.81	2.34	0.00
Prolyl endopeptidase OS = Mus musculus GN = Prep PE = 2 SV = 1	PPCE_MOUSE	81 kDa	3.75	0.00	5.57
Propionyl-CoA carboxylase alpha chain, mitochondrial OS = Mus musculus GN = Pcca PE = 2 SV = 2	PCCA_MOUSE	80 kDa	0.00	0.00	2.79
Prostaglandin E synthase 3 OS = Mus musculus GN = Ptges3 PE = 1 SV = 1	TEBP_MOUSE	19 kDa	3.75	3.50	3.71
Proteasome subunit alpha type-2 OS = Mus musculus GN = Psma2 PE = 1 SV = 3	PSA2_MOUSE	26 kDa	6.56	3.50	5.57
Protein bassoon OS = Mus musculus GN = Bsn PE = 1 SV = 4	BSN_MOUSE	419 kDa	9.37	7.01	13.93
Protein disulfide-isomerase A3 OS = Mus musculus GN = Pdia3 PE = 1 SV = 2	PDIA3_MOUSE	57 kDa	1.87	0.00	2.79
Protein disulfide-isomerase OS = Mus musculus GN = P4hb PE = 1 SV = 2	PDIA1_MOUSE	57 kDa	1.87	4.67	3.71
Protein kinase C and casein kinase substrate in neurons protein 1 OS = Mus musculus GN = Pacsin1 PE = 1 SV = 1	PACN1_MOUSE	51 kDa	22.50	37.38	27.85
Protein kinase C gamma type OS = Mus musculus GN = Prkcg PE = 1 SV = 1	KPCG_MOUSE	78 kDa	8.44	1.17	5.57
Protein NDRG2 OS = Mus musculus GN = Ndrg2 PE = 1 SV = 1	NDRG2_MOUSE	41 kDa	3.75	5.84	2.79
Protein phosphatase 1 regulatory subunit 29 OS = Mus musculus GN = Elfn2 PE = 1 SV = 1	PPR29_MOUSE	90 kDa	10.31	2.34	7.43
Protein phosphatase 1E OS = Mus musculus GN = Ppm1e PE = 1 SV = 2	PPM1E_MOUSE	83 kDa	2.81	0.00	3.71
Putative ATP-dependent RNA helicase Pl10 OS = Mus musculus GN = D1Pas1 PE = 1 SV = 1	DDX3L_MOUSE (+2)	73 kDa	2.81	2.34	2.79
Putative tyrosine-protein phosphatase auxilin OS = Mus musculus GN = Dnajc6 PE = 2 SV = 2	AUXI_MOUSE	102 kDa	1.87	2.34	4.64

Table S1. *Cont.*

Protein Descriptions	Protein Name (Swissprot)	Molecular Weight	Quantitative Value		
			0 cGy	0.1 Gy	4 Gy
Pyridoxal kinase OS = Mus musculus GN = Pdxk PE = 1 SV = 1	PDXK_MOUSE	35 kDa	10.31	1.17	10.21
Pyruvate carboxylase, mitochondrial OS = Mus musculus GN = Pc PE = 1 SV = 1	PYC_MOUSE	130 kDa	3.75	7.01	7.43
Pyruvate dehydrogenase E1 component subunit beta, mitochondrial OS = Mus musculus GN = Pdhb PE = 1 SV = 1	ODPB_MOUSE	39 kDa	7.50	7.01	6.50
Pyruvate kinase isozymes M1/M2 OS = Mus musculus GN = Pkm PE = 1 SV = 4	KPYM_MOUSE	58 kDa	21.56	33.88	19.50
Rab GDP dissociation inhibitor alpha OS = Mus musculus GN = Gdi1 PE = 1 SV = 3	GDIA_MOUSE	51 kDa	11.25	5.84	7.43
Rabphilin-3A OS = Mus musculus GN = Rph3a PE = 1 SV = 2	RP3A_MOUSE	75 kDa	3.75	4.67	3.71
RAC-gamma serine/threonine-protein kinase OS = Mus musculus GN = Akt3 PE = 1 SV = 1	AKT3_MOUSE	56 kDa	0.00	4.67	3.71
Ras-related C3 botulinum toxin substrate 1 OS = Mus musculus GN = Rac1 PE = 1 SV = 1	RAC1_MOUSE	21 kDa	3.75	11.68	7.43
Ras-related protein Rab-10 OS = Mus musculus GN = Rab10 PE = 1 SV = 1	RAB10_MOUSE	23 kDa	1.87	2.34	2.79
Ras-related protein Rab-21 OS = Mus musculus GN = Rab21 PE = 1 SV = 4	RAB21_MOUSE	24 kDa	1.87	0.00	3.71
Ras-related protein Rab-3A OS = Mus musculus GN = Rab3a PE = 1 SV = 1	RAB3A_MOUSE	25 kDa	11.25	9.35	12.07
Reticulon-4 OS = Mus musculus GN = Rtn4 PE = 1 SV = 2	RTN4_MOUSE	127 kDa	7.50	10.51	8.36
Rho-related GTP-binding protein RhoB OS = Mus musculus GN = Rhob PE = 1 SV = 1	RHOB_MOUSE	22 kDa	6.56	9.35	9.28
Ribosomal protein S6 kinase alpha-3 OS = Mus musculus GN = Rps6ka3 PE = 1 SV = 2	KS6A3_MOUSE	84 kDa	3.75	4.67	3.71
Ryanodine receptor 2 OS = Mus musculus GN = Ryr2 PE = 1 SV = 1	RYR2_MOUSE	565 kDa	3.75	1.17	5.57
Saccharopine dehydrogenase-like oxidoreductase OS = Mus musculus GN = Sccpdh PE = 2 SV = 1	SCPDL_MOUSE	47 kDa	2.81	0.00	0.93
Selenium-binding protein 1 OS = Mus musculus GN = Selenbp1 PE = 1 SV = 2	SBP1_MOUSE (+1)	53 kDa	4.69	0.00	2.79
Septin-11 OS = Mus musculus GN = Sept11 PE = 1 SV = 4	SEP11_MOUSE	50 kDa	1.87	0.00	4.64
Septin-5 OS = Mus musculus GN = Sept5 PE = 1 SV = 2	SEPT5_MOUSE	43 kDa	1.87	4.67	6.50
Septin-7 OS = Mus musculus GN = Sept7 PE = 1 SV = 1	SEPT7_MOUSE	51 kDa	1.87	2.34	3.71
Serine racemase OS = Mus musculus GN = Srr PE = 1 SV = 1	SRR_MOUSE	36 kDa	3.75	0.00	3.71

Table S1. *Cont.*

Protein Descriptions	Protein Name (Swissprot)	Molecular Weight	Quantitative Value		
			0 cGy	0.1 Gy	4 Gy
Serine/threonine-protein kinase DCLK1 OS = Mus musculus GN = Dclk1 PE = 1 SV = 1	DCLK1_MOUSE	84 kDa	2.81	0.00	4.64
Serine/threonine-protein phosphatase 2A 65 kDa regulatory subunit A alpha isoform OS = Mus musculus GN = Ppp2r1a PE = 1 SV = 3	2AAA_MOUSE	65 kDa	7.50	7.01	8.36
Serine/threonine-protein phosphatase 2B catalytic subunit alpha isoform OS = Mus musculus GN = Ppp3ca PE = 1 SV = 1	PP2BA_MOUSE	59 kDa	18.75	26.87	18.57
Serine/threonine-protein phosphatase PP1-alpha catalytic subunit OS = Mus musculus GN = Ppp1ca PE = 1 SV = 1	PP1A_MOUSE (+1)	38 kDa	4.69	18.69	3.71
Serine--tRNA ligase, cytoplasmic OS = Mus musculus GN = Sars PE = 2 SV = 3	SYSC_MOUSE	58 kDa	2.81	2.34	1.86
Serum albumin OS = Mus musculus GN = Alb PE = 1 SV = 3	ALBU_MOUSE	69 kDa	17.81	11.68	15.78
S-formylglutathione hydrolase OS = Mus musculus GN = Esd PE = 2 SV = 1	ESTD_MOUSE	31 kDa	17.81	14.02	12.07
SH3-containing GRB2-like protein 3-interacting protein 1 OS = Mus musculus GN = Sgip1 PE = 1 SV = 1	SGIP1_MOUSE	86 kDa	5.62	2.34	3.71
Sideroflexin-3 OS = Mus musculus GN = Sfxn3 PE = 1 SV = 1	SFXN3_MOUSE	35 kDa	5.62	8.18	7.43
Sideroflexin-5 OS = Mus musculus GN = Sfxn5 PE = 1 SV = 2	SFXN5_MOUSE	37 kDa	5.62	7.01	8.36
Small nuclear ribonucleoprotein Sm D3 OS = Mus musculus GN = Snrpd3 PE = 1 SV = 1	SMD3_MOUSE	14 kDa	7.50	7.01	7.43
Sodium- and chloride-dependent GABA transporter 3 OS = Mus musculus GN = Slc6a11 PE = 1 SV = 2	S6A11_MOUSE (+1)	70 kDa	1.87	0.00	2.79
Sodium/potassium-transporting ATPase subunit alpha-1 OS = Mus musculus GN = Atp1a1 PE = 1 SV = 1	AT1A1_MOUSE	113 kDa	6.56	2.34	6.50
Sodium/potassium-transporting ATPase subunit alpha-2 OS = Mus musculus GN = Atp1a2 PE = 1 SV = 1	AT1A2_MOUSE	112 kDa	8.44	1.17	8.36
Sodium/potassium-transporting ATPase subunit alpha-3 OS = Mus musculus GN = Atp1a3 PE = 1 SV = 1	AT1A3_MOUSE	112 kDa	28.12	14.02	35.28
Sodium/potassium-transporting ATPase subunit beta-1 OS = Mus musculus GN = Atp1b1 PE = 1 SV = 1	AT1B1_MOUSE	35 kDa	25.31	120.32	20.43
Sodium-dependent neutral amino acid transporter SLC6A17 OS = Mus musculus GN = Slc6a17 PE = 1 SV = 1	S6A17_MOUSE	81 kDa	7.50	9.35	8.36

Table S1. *Cont.*

Protein Descriptions	Protein Name (Swissprot)	Molecular Weight	Quantitative Value		
			0 cGy	0.1 Gy	4 Gy
Sodium-driven chloride bicarbonate exchanger OS = Mus musculus GN = Slc4a10 PE = 1 SV = 2	S4A10_MOUSE	126 kDa	3.75	5.84	4.64
Solute carrier family 12 member 5 OS = Mus musculus GN = Slc12a5 PE = 1 SV = 2	S12A5_MOUSE	126 kDa	0.94	4.67	5.57
Sorbin and SH3 domain-containing protein 1 OS = Mus musculus GN = Sorbs1 PE = 1 SV = 2	SRBS1_MOUSE	143 kDa	0.00	2.34	3.71
Sorting and assembly machinery component 50 homolog OS = Mus musculus GN = Samm50 PE = 1 SV = 1	SAM50_MOUSE	52 kDa	1.87	5.84	1.86
Spectrin alpha chain, brain OS = Mus musculus GN = Sptan1 PE = 1 SV = 4	SPTA2_MOUSE	?	36.56	31.54	38.06
Spectrin beta chain, non-erythrocytic 1 OS = Mus musculus GN = Sptbn1 PE = 1 SV = 2	SPTB2_MOUSE	274 kDa	19.69	16.35	17.64
Spliceosome RNA helicase Ddx39b OS = Mus musculus GN = Ddx39b PE = 1 SV = 1	DX39B_MOUSE	49 kDa	15.00	15.19	18.57
Succinate dehydrogenase [ubiquinone] flavoprotein subunit, mitochondrial OS = Mus musculus GN = Sdha PE = 1 SV = 1	DHSA_MOUSE	73 kDa	4.69	4.67	8.36
Succinate dehydrogenase cytochrome b560 subunit, mitochondrial OS = Mus musculus GN = Sdhc PE = 2 SV = 1	C560_MOUSE	18 kDa	4.69	5.84	3.71
Succinyl-CoA ligase [ADP-forming] subunit beta, mitochondrial OS = Mus musculus GN = Sucla2 PE = 1 SV = 2	SUCB1_MOUSE	50 kDa	3.75	4.67	3.71
Superoxide dismutase [Mn], mitochondrial OS = Mus musculus GN = Sod2 PE = 1 SV = 3	SODM_MOUSE	25 kDa	8.44	16.35	7.43
Synapsin-1 OS = Mus musculus GN = Syn1 PE = 1 SV = 2	SYN1_MOUSE	74 kDa	7.50	5.84	10.21
Synapsin-2 OS = Mus musculus GN = Syn2 PE = 1 SV = 2	SYN2_MOUSE	63 kDa	14.06	9.35	12.07
Synaptic vesicle glycoprotein 2A OS = Mus musculus GN = Sv2a PE = 1 SV = 1	SV2A_MOUSE	83 kDa	9.37	18.69	7.43
Synaptic vesicle glycoprotein 2B OS = Mus musculus GN = Sv2b PE = 1 SV = 1	SV2B_MOUSE	77 kDa	14.06	16.35	13.00
Synaptojanin-1 OS = Mus musculus GN = Synj1 PE = 1 SV = 3	SYNJ1_MOUSE	173 kDa	3.75	4.67	3.71
Synaptophysin OS = Mus musculus GN = Syp PE = 1 SV = 2	SYPH_MOUSE	34 kDa	2.81	0.00	1.86
Synaptotagmin-1 OS = Mus musculus GN = Syt1 PE = 1 SV = 1	SYT1_MOUSE	47 kDa	44.06	46.73	45.49
Syntaxin-binding protein 1 OS = Mus musculus GN = Stxbp1 PE = 1 SV = 2	STXB1_MOUSE	68 kDa	9.37	21.03	13.93

Table S1. *Cont.*

Protein Descriptions	Protein Name (Swissprot)	Molecular Weight	Quantitative Value		
			0 cGy	0.1 Gy	4 Gy
T-complex protein 1 subunit beta OS = Mus musculus GN = Cct2 PE = 1 SV = 4	TCPB_MOUSE	57 kDa	1.87	2.34	2.79
T-complex protein 1 subunit eta OS = Mus musculus GN = Cct7 PE = 1 SV = 1	TCPH_MOUSE	60 kDa	1.87	0.00	2.79
T-complex protein 1 subunit theta OS = Mus musculus GN = Cct8 PE = 1 SV = 3	TCPQ_MOUSE	60 kDa	3.75	4.67	5.57
Tenascin-R OS = Mus musculus GN = Tnr PE = 1 SV = 2	TENR_MOUSE	150 kDa	7.50	10.51	13.00
Thioredoxin-dependent peroxide reductase, mitochondrial OS = Mus musculus GN = Prdx3 PE = 1 SV = 1	PRDX3_MOUSE	28 kDa	2.81	0.00	1.86
Thy-1 membrane glycoprotein OS = Mus musculus GN = Thy1 PE = 1 SV = 1	THY1_MOUSE	18 kDa	19.69	28.04	24.14
Toll-interacting protein OS = Mus musculus GN = Tollip PE = 1 SV = 1	TOLIP_MOUSE	30 kDa	2.81	2.34	3.71
Transaldolase OS = Mus musculus GN = Taldo1 PE = 1 SV = 2	TALDO_MOUSE	37 kDa	2.81	2.34	2.79
Transforming protein RhoA OS = Mus musculus GN = Rhoa PE = 1 SV = 1	RHOA_MOUSE (+1)	22 kDa	10.31	15.19	17.64
Transgelin OS = Mus musculus GN = Tagln PE = 1 SV = 3	TAGL_MOUSE	23 kDa	4.69	1.17	2.79
Transgelin-3 OS = Mus musculus GN = Tagln3 PE = 1 SV = 1	TAGL3_MOUSE	22 kDa	1.87	0.00	3.71
Trifunctional enzyme subunit beta, mitochondrial OS = Mus musculus GN = Hadhb PE = 1 SV = 1	ECHB_MOUSE	51 kDa	2.81	2.34	1.86
Triosephosphate isomerase OS = Mus musculus GN = Tpi1 PE = 1 SV = 4	TPIS_MOUSE	32 kDa	22.50	11.68	21.35
Tubulin alpha-1A chain OS = Mus musculus GN = Tuba1a PE = 1 SV = 1	TBA1A_MOUSE	50 kDa	114.36	110.97	115.12
Tubulin alpha-1B chain OS = Mus musculus GN = Tuba1b PE = 1 SV = 2	TBA1B_MOUSE	50 kDa	127.48	115.64	116.05
Tubulin alpha-4A chain OS = Mus musculus GN = Tuba4a PE = 1 SV = 1	TBA4A_MOUSE	50 kDa	119.99	110.97	112.34
Tubulin beta-2A chain OS = Mus musculus GN = Tubb2a PE = 1 SV = 1	TBB2A_MOUSE	50 kDa	218.41	212.60	126.26
Tubulin beta-3 chain OS = Mus musculus GN = Tubb3 PE = 1 SV = 1	TBB3_MOUSE	50 kDa	41.25	22.19	37.14
Tubulin beta-4 chain OS = Mus musculus GN = Tubb4 PE = 1 SV = 3	TBB4_MOUSE	?	222.16	195.08	113.26
Twinfilin-1 OS = Mus musculus GN = Twf1 PE = 1 SV = 2	TWF1_MOUSE	40 kDa	1.87	0.00	1.86
Ubiquitin carboxyl-terminal hydrolase 5 OS = Mus musculus GN = Usp5 PE = 1 SV = 1	UBP5_MOUSE	96 kDa	3.75	1.17	3.71

Table S1. *Cont.*

Protein Descriptions	Protein Name (Swissprot)	Molecular Weight	Quantitative Value		
			0 cGy	0.1 Gy	4 Gy
Ubiquitin carboxyl-terminal hydrolase isozyme L1 OS = Mus musculus GN = Uchl1 PE = 1 SV = 1	UCHL1_MOUSE	25 kDa	7.50	0.00	6.50
Ubiquitin-conjugating enzyme E2 N OS = Mus musculus GN = Ube2n PE = 1 SV = 1	UBE2N_MOUSE	17 kDa	3.75	2.34	3.71
Ubiquitin-like modifier-activating enzyme 1 OS = Mus musculus GN = Uba1 PE = 1 SV = 1	UBA1_MOUSE	118 kDa	7.50	10.51	7.43
UMP-CMP kinase OS = Mus musculus GN = Cmpk1 PE = 1 SV = 1	KCY_MOUSE	22 kDa	1.87	2.34	3.71
Vacuolar protein sorting-associated protein 26A OS = Mus musculus GN = Vps26a PE = 2 SV = 1	VP26A_MOUSE	38 kDa	5.62	4.67	6.50
Vacuolar protein sorting-associated protein 35 OS = Mus musculus GN = Vps35 PE = 1 SV = 1	VPS35_MOUSE	92 kDa	1.87	1.17	2.79
Vesicle-fusing ATPase OS = Mus musculus GN = Nsf PE = 1 SV = 2	NSF_MOUSE	83 kDa	5.62	3.50	9.28
Vimentin OS = Mus musculus GN = Vim PE = 1 SV = 3	VIME_MOUSE	54 kDa	9.37	5.84	3.71
Voltage-dependent anion-selective channel protein 1 OS = Mus musculus GN = Vdac1 PE = 1 SV = 3	VDAC1_MOUSE	32 kDa	14.06	7.01	9.28
Voltage-dependent anion-selective channel protein 2 OS = Mus musculus GN = Vdac2 PE = 1 SV = 2	VDAC2_MOUSE	32 kDa	3.75	0.00	4.64
Voltage-dependent anion-selective channel protein 3 OS = Mus musculus GN = Vdac3 PE = 1 SV = 1	VDAC3_MOUSE	31 kDa	2.81	1.17	1.86
V-type proton ATPase 116 kDa subunit a isoform 1 OS = Mus musculus GN = Atp6v0a1 PE = 1 SV = 3	VPP1_MOUSE	96 kDa	16.87	16.35	21.35
V-type proton ATPase catalytic subunit A OS = Mus musculus GN = Atp6v1a PE = 1 SV = 2	VATA_MOUSE	68 kDa	27.18	30.37	30.64
V-type proton ATPase subunit B, brain isoform OS = Mus musculus GN = Atp6v1b2 PE = 1 SV = 1	VATB2_MOUSE	57 kDa	5.62	9.35	9.28
V-type proton ATPase subunit C 1 OS = Mus musculus GN = Atp6v1c1 PE = 1 SV = 4	VATC1_MOUSE	44 kDa	9.37	12.85	3.71
V-type proton ATPase subunit E 1 OS = Mus musculus GN = Atp6v1e1 PE = 1 SV = 2	VATE1_MOUSE (+1)	26 kDa	6.56	8.18	8.36
V-type proton ATPase subunit H OS = Mus musculus GN = Atp6v1h PE = 1 SV = 1	VATH_MOUSE	56 kDa	6.56	7.01	6.50
WD repeat-containing protein 1 OS = Mus musculus GN = Wdr1 PE = 1 SV = 3	WDR1_MOUSE	66 kDa	6.56	5.84	4.64
WD repeat-containing protein 7 OS = Mus musculus GN = Wdr7 PE = 1 SV = 3	WDR7_MOUSE	163 kDa	13.12	9.35	13.00

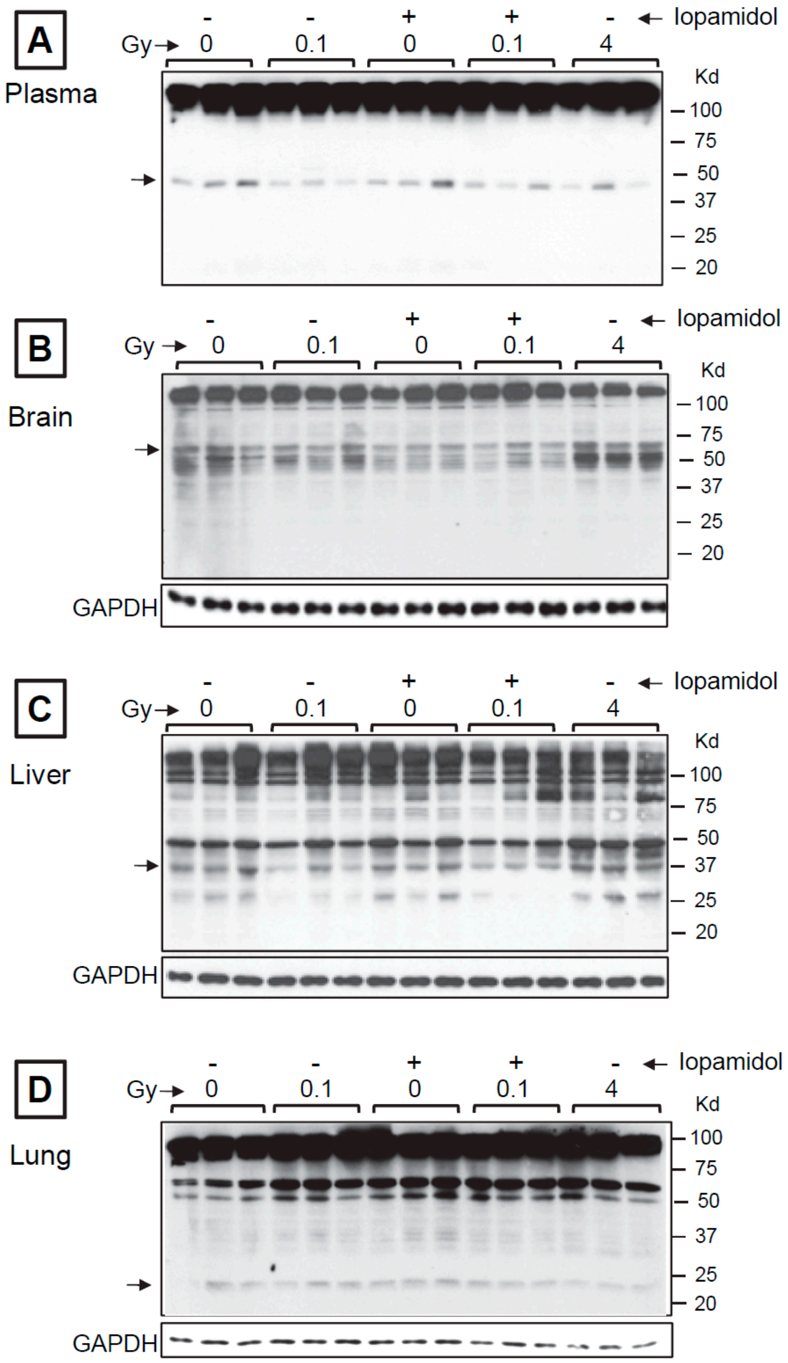

Figure S1. Modulation of global *S*-nitrosylation in organs of mice exposed to ionizing radiation in the presence or absence of iodine contrast agent. Western blot analyses, following biotin-switch assay, of nitrosylated proteins from plasma (**A**), brain (**B**), liver (**C**), and lung (**D**) of C57Bl/6J mice exposed 13 days earlier to ^{137}Cs γ-rays in presence (+) or absence (−) of iopamidol. Proteins from mouse organs were freshly extracted and subjected to the biotin switch assay. The biotinylated proteins were detected with anti-biotin antibody. Protein aliquots (15 µg), before enrichment, were used as input standard and the expression level of GAPDH was used as loading control. In case of plasma from circulating blood, staining of the membrane with Ponceau S Red (not shown) indicated equal loading.

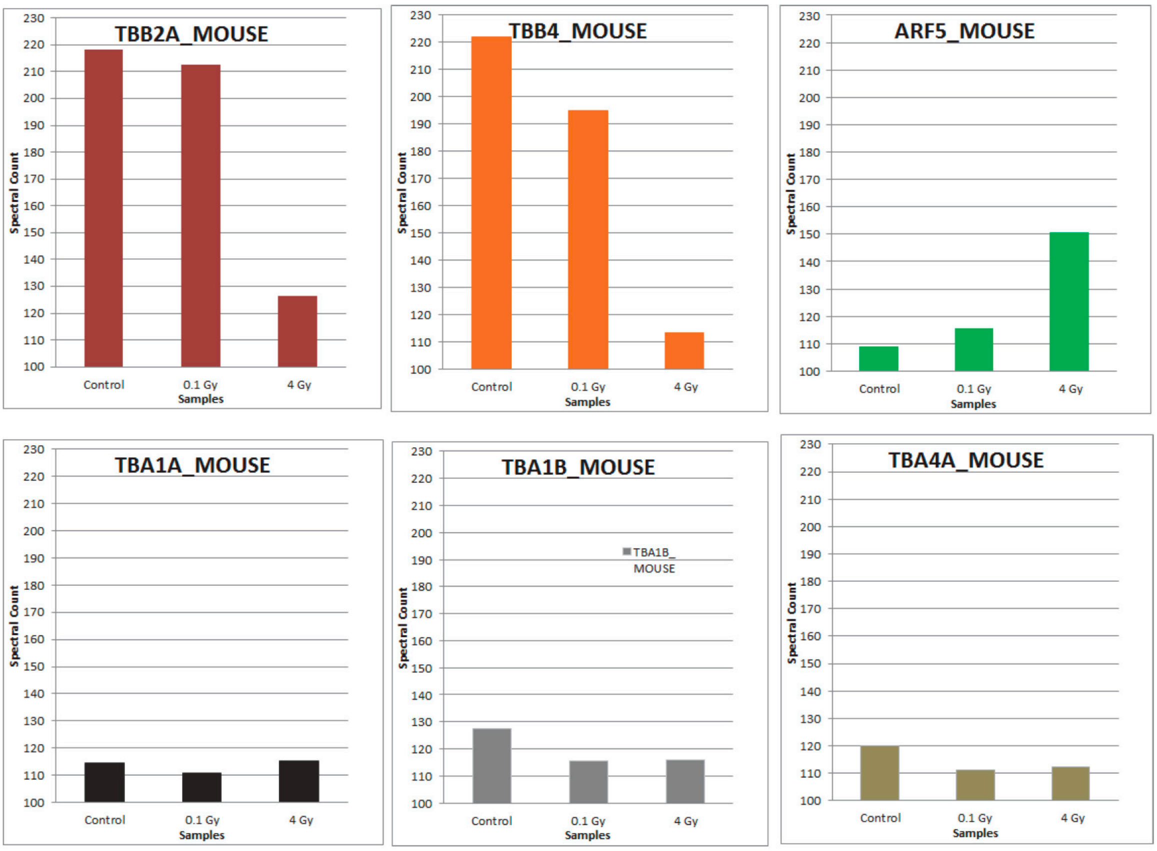

Figure S2. Differential effect of radiation dose on *S*-nitrosylation level. Bar chart comparisons of spectral counts of SNO proteins (proteins with spectral counts above 80) showing differential modulation in *S*-nitrosylation levels in brains of mice exposed to 0, 0.1 or 4 Gy of ^{137}Cs γ-rays.
